# Soft Robotics and Advanced Technologies for Minimally Invasive Bioprinting: The Future of Internal Organ Repair

**DOI:** 10.1002/advs.202523532

**Published:** 2026-03-19

**Authors:** Duc Tu Vu, Nhu An Phan, Sy Trung Ngo, Minh Tri Phan, Thanh‐An Truong, Chi Cong Nguyen, Phuoc Thien Phan, Hoang‐Phuong Phan, Thanh Nho Do, Mai Thanh Thai

**Affiliations:** ^1^ College of Engineering & Computer Science VinUniversity Hanoi Vietnam; ^2^ VinUni‐Illinois Smart Health Center VinUniversity Hanoi Vietnam; ^3^ School of Engineering and Built Environment Queensland Quantum and Advanced Technologies Research Institute (QUATRI) Griffith University Queensland Australia; ^4^ School of Mechanical and Manufacturing Engineering University of New South Wales Sydney New South Wales Australia; ^5^ School of Biomedical Engineering Faculty of Engineering UNSW Sydney Sydney New South Wales Australia

**Keywords:** flexible actuation, in situ bioprinting, minimally invasive surgery, robotic‐assisted bioprinting, soft robotics, tissue regeneration

## Abstract

Bioprinting, first proposed in the 1980s for ex vivo tissue fabrication, has evolved into a cornerstone of regenerative medicine. Conventional approaches rely on printing tissues outside the body for later implantation but are limited by geometric mismatch, construct fragility, and invasive surgery. In situ bioprinting addresses these limitations by depositing cells and biomaterials directly at defect sites, enabling patient‐specific repair and improved tissue integration. Building on this paradigm, Minimally Invasive Bioprinting (MIB) targets internal organ regeneration through small incisions or natural orifices. This review defines a technological roadmap from handheld bioprinting tools to advanced MIB systems, identifying soft robotics as the primary hardware enabler for navigation within confined anatomical environments. We examine essential technology pillars for MIB, including soft actuation, sensing, real‐time imaging, computational modeling, intelligent control, and bioink engineering. The integration of emerging approaches such as artificial intelligence, four‐dimensional bioprinting, and organ‐on‐a‐chip platforms is discussed for enhancing autonomy, adaptability, and functional outcomes. Finally, we evaluate key translational challenges, including safety, scalability, and reproducibility, and outline regulatory considerations for clinical implementation. Overall, integrating soft robotic mechanisms with in situ bioprinting is critical for achieving safe, high‐fidelity, patient‐specific internal organ repair in minimally invasive clinical settings worldwide for future practice applications.

## Graphical abstract

This review examines the evolution of bioprinting toward minimally invasive in situ strategies for internal organ regeneration. It defines the technological roadmap from handheld systems to advanced minimally invasive bioprinting platforms, positioning soft robotics as a core enabler. Key technological pillars, emerging integrations, translational barriers, and regulatory considerations are systematically analyzed.

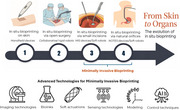

## 1 Introduction: Toward Precision Organ Repair Through Minimally Invasive Bioprinting and Soft Robotics

Repairing damaged tissues and internal organs remains one of the greatest challenges in modern medicine [1]. The human body is composed of complex, curved, and sensitive anatomical structures, making it extremely difficult to access and repair deep tissue defects without causing additional harm. Traditional surgical interventions often require large, invasive incisions to reach these sites, which can increase patient trauma, elevate the risk of post‐operative complications, and prolong hospital stays and recovery times [2, 3]. These challenges have driven the development of alternative therapeutic strategies that aim to restore function while minimizing surgical invasiveness.

One of the most promising technologies to address these limitations is bioprinting, a rapidly evolving technique that enables the fabrication of tissue‐like structures using living cells and biomaterials. By mimicking the native architecture of biological tissues, bioprinting holds great potential for tissue regeneration and even whole‐organ fabrication [4, 5]. Traditionally, bioprinting has been performed outside the body, where tissue constructs are printed in controlled environments and later surgically implanted into the patient. This process is still limited by several drawbacks, including poor geometric matching between the pre‐fabricated construct and the actual defect site, the risk of damaging fragile constructs during handling and implantation, and the need for invasive surgeries to position the printed tissue at the defect site [3, 6].

To overcome these challenges, researchers have introduced in situ bioprinting. This novel approach enables the direct deposition of bioinks and living cells onto or within the target tissue inside the body. By printing directly at the defect site, this method enhances geometric accuracy, reduces the need for handling, and improves integration with the surrounding biological environment. To truly realize these benefits, the goal is to deploy these bioprinting systems using a Minimally Invasive Surgery (MIS) approach, which is known to minimize patient trauma, accelerate recovery times, and alleviate post‐operative pain compared to traditional open surgery [7, 8]. A key technological enabler of this approach is soft robotics [3, 9]. Unlike rigid robotic systems, soft robotic devices, made from flexible, biocompatible materials, can safely adapt to the body's complex geometry, making them ideal for performing delicate tasks with minimal tissue disruption [3, 10, 11].

**FIGURE 1:**
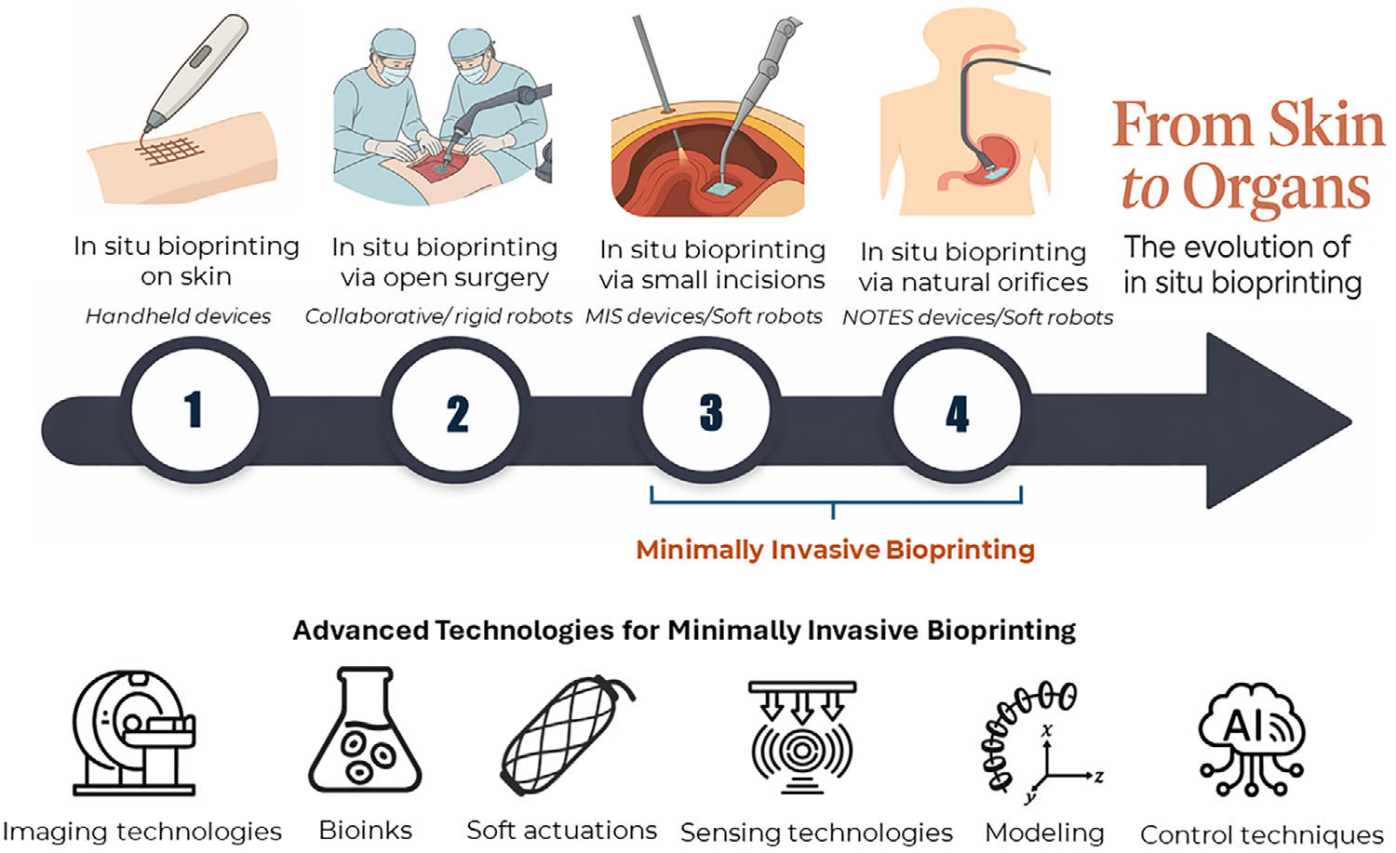
Conceptual framework illustrating the evolution of in situ bioprinting from surface‐level repair (handheld approaches) to open‐surgery and robot‐assisted platforms, culminating in minimally invasive internal organ bioprinting via MIS/NOTES devices and soft‐robotic systems, alongside the core enabling technology modules (imaging, bioinks, soft actuations, sensing, modeling, and control).

To visualize the technological trajectory of this field, Figure 1 presents a conceptual roadmap outlining the evolution of in situ bioprinting technologies across four developmental stages, each representing a progression toward greater anatomical access with reduced invasiveness. **Stage 1** focuses on surface‐level bioprinting, where bioinks are applied directly onto external wounds (typically skin), commonly using handheld devices, a 3‐axis robotic bioprinter [12, 13, 14]. This portability makes them ideal for urgent scenarios, allowing for rapid deployment in emergency settings to accelerate the healing and regeneration of extensive skin defects. **Stage 2** involves in situ bioprinting via open surgery, primarily utilizing collaborative robots or rigid robotic platforms [12, 13, 15]. These systems enable the direct reconstruction of deeper tissues, such as cartilage or internal organs, with high stability. However, due to their bulky design and rigid kinematics, they require a substantial operational workspace, limiting their application exclusively to traditional open surgical procedures.

**Stage 3** advances to in situ bioprinting via small incisions, primarily employing tools for minimally invasive surgery, for example, laparoscopic instruments or modern platforms like the da Vinci and RAVEN surgery system [16, 17]. These systems typically utilize instruments with long, slender shafts and integrated cameras for visual guidance. Access to internal organs is achieved through small incisions in the abdominal wall, significantly reducing surgical trauma compared to open procedures. Since these platforms are originally designed for surgical tasks such as resection or suturing, they have the potential to be retrofitted with custom printing head modules mounted onto their distal end‐effectors to enable bioprinting capabilities. Recently, this stage also encompasses the emerging application of flexible platforms based on soft robotics [10, 18]. These soft robotic solutions offer a promising avenue to address the limitations of rigid instrumentation, primarily offering key advantages such as flexible navigation to access confined target sites and enhanced biosafety due to their inherent mechanical compliance.

**Stage 4**, the most futuristic and least invasive, explores bioprinting through natural orifices using soft robotics. This approach builds upon the principles of Natural Orifice Transluminal Endoscopic Surgery (NOTES), a technique that allows flexible instruments to access internal organs via the mouth, anus, or vagina. By navigating into the body's intricate and curved pathways without external incisions, NOTES lays the groundwork for advanced, incision‐free interventions. Several robotic platforms have already been developed for NOTES procedures, including the MASTER system, Medrobotics Flex System, Viacath, and R‐Scope [19, 20, 21, 22]. Similar to MIS tools in stage 3, these surgical robots could theoretically be equipped with a bioprinting nozzle module at their tips. However, as this concept remains in its infancy, the integration of bioprinting modules onto these existing rigid NOTES platforms remains largely unexplored. Instead, recent studies have focused on developing flexible robots based on soft robotics, specifically for in situ bioprinting via natural orifices [10, 23]. These emerging devices demonstrate the great potential of soft robotics, offering a highly promising solution for internal organ repair.

Together, these approaches, ranging from laparoscopic access in Stage 3 to natural orifice navigation in Stage 4, are unified under the term Minimally Invasive Bioprinting (MIB). Executing precise biofabrication within the confined and dynamic environments of the human body demands a synergistic integration of enabling technologies. As illustrated in the bottom panel of Figure 1, the MIB ecosystem is underpinned by six core technology pillars: imaging technologies, bioink preparation strategies, soft actuation, sensing technologies, modeling, and advanced control techniques. A comprehensive analysis of each pillar is presented in the following sections, highlighting the current state‐of‐the‐art and the critical challenges that must be addressed to realize next‐generation bioprinting systems.

To date, the existing literature has not yet synthesized the convergence of these domains. Reviews on bioprinting have predominantly concentrated on material science aspects, such as bioink formulations and crosslinking mechanisms [24, 25, 26]. Regarding in situ bioprinting applications, discussions are largely restricted to cutaneous wound healing or open surgical procedures using handheld devices or conventional robotic arms [5, 27, 28, 29, 30]. While the concept of MIB has been acknowledged in some recent works, it is discussed as a future perspective or conceptual vision rather than a subject of in‐depth technical analysis [9, 31, 32, 33].

Conversely, biomedical soft robotics reviews have extensively surveyed broad clinical applications [34, 35, 36, 37, 38], or specific domains such as rehabilitation [39, 40, 41], targeted drug delivery [42], prosthetics [43], and artificial organs [44]. The literature most closely related to our topic includes reviews on soft robotics for MIS [3, 45, 46, 47, 48]. However, these primarily emphasize sensing, modeling, and control for surgical manipulation and do not directly address the requirements of integrating soft robotic platforms with bioprinting functions (e.g., deposition, stabilization, and closed‐loop execution).

As a result, a clear gap remains: there is still no comprehensive review that systematically analyzes the intersection of soft robotics and MIB. Importantly, we contend that the absence of purpose‐built robotic delivery platforms is a major barrier to achieving MIB, especially for deep‐tissue access, where soft robotic continuum systems offer a uniquely safe and compliant solution for navigation in confined anatomy. This review bridges that gap by providing a dedicated, technology‐focused analysis of the enabling pillars required for high‐fidelity in situ tissue regeneration in minimally invasive settings, highlighting the transformative potential of soft robotics as a foundational hardware enabler for MIB.

To substantiate this potential and provide a structured pathway for its realization, the present review examines the state of the art at the intersection of soft robotics and in situ bioprinting, explicitly addressing five core objectives: (1) To delineate the evolutionary pathway of in situ bioprinting, highlighting the transition toward Minimally Invasive Bioprinting as a transformative approach for internal organ repair; (2) To establish the pivotal role of soft robotics as the essential hardware enabler that allows bioprinting devices to navigate and operate safely within confined anatomical environments; (3) To comprehensively analyze the advanced technology pillars, including imaging, bioinks, soft actuation, sensing, modeling, and control, that constitute the foundation of functional MIB systems; (4) To explore the integration of emerging future technologies, such as 4D bioprinting, organ‐on‐a‐chip models, and artificial intelligence, in enhancing the autonomy and functionality of next‐generation bioprinters; and (**5**) To critically evaluate the current technical and regulatory barriers, providing a structured discussion on the challenges and pathways toward clinical translation.

## 2 The Role of Soft Robotics in Medical Innovation as a Foundation for MIS and MIB

Before examining soft‐robotic bioprinting systems, we first summarize how soft robotics has become a core enabling technology for minimally invasive surgery (MIS). The same properties, compliance, safe tissue interaction, and navigation through tortuous anatomy also make soft robotics the natural technological foundation for MIB, where a flexible robotic delivery platform is required to position and operate a printhead at internal defect sites.

Soft robotics is an emerging field focused on the design and development of robots made from flexible, deformable, and often stretchable materials, closely mimicking the properties of biological tissues. Unlike conventional rigid robots, which are typically constructed from metals such as steel or aluminum [49], soft robots are composed of materials like silicones, elastomers, hydrogels, and other polymers. These materials allow for bending, twisting, and compression in a way that is safe for interaction with the human body [50, 51]. These compliant mechanical characteristics make soft robots particularly suitable for biomedical applications, where safe, adaptive interaction with delicate and irregularly shaped biological structures is essential.

The human body is largely composed of soft, dynamic tissues—muscles, vessels, and organs—that can be easily damaged by rigid instruments [49]. Soft robots, by contrast, are inherently compliant and can conform to biological surfaces, enabling them to operate safely within confined anatomical spaces. This flexibility reduces the risk of tissue damage and facilitates safer, more precise interventions in clinical settings [5].

**FIGURE 2:**
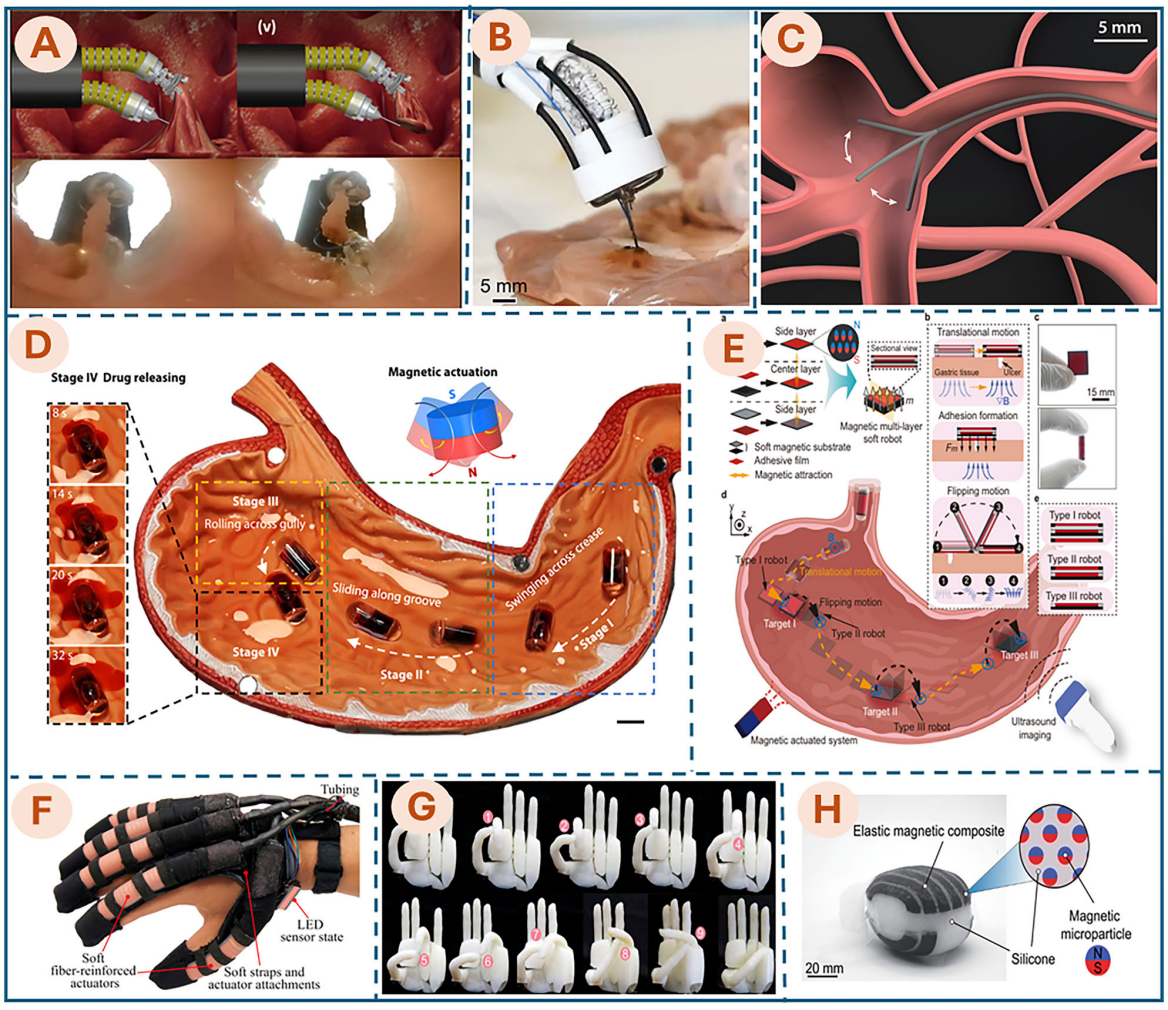
Emerging applications of soft robotics in medicine. (A) Bioinspired soft robot system for MIS. Reproduced with permission from ref. [52]. Copyright 2025, AAAS. (B) F3DB soft robotics system. Reproduced with permission from ref. [10]. Copyright 2023, John Wiley and Sons. (C) Ferromagnetic soft continuum robots. Reproduced with permission from ref. [53]. Copyright 2019, AAAS. (D) MagCaps drug delivery device. Reproduced with permission from ref. [54]. Copyright 2019, AAAS. (E) Magnetic multi‐layer soft robot. Reproduced with permission from ref. [55]. Copyright 2024, Springer Nature. (F) Soft robotic glove. Reproduced with permission from ref. [56]. Copyright 2015, Elsevier. (G) Soft Robotic Hand. Adapted with permission from ref. [57]. Copyright 2019, IEEE. (H) Soft‐robotics bladder. Reproduced with permission from ref. [58]. Copyright 2022, AAAS.

In recent years, soft robotics has shown remarkable promise across a wide range of biomedical domains, as shown in Figure 2. In MIS, soft robotic tools have been developed to navigate through tight or curved anatomical pathways, enabling access to target sites while minimizing tissue trauma and patient recovery time [3]. They also support inner curing by guiding tiny robots to specific areas for tasks such as vessel occlusion or local therapeutic delivery. In tissue engineering, soft robotic arms can precisely place cells and biomaterials, enabling the delicate construction and repair of tissues [10, 59]. For drug delivery, miniaturized soft robots can transport and release medications directly at diseased sites, improving therapeutic outcomes while minimizing side effects [60]. In rehabilitation, wearable soft robotic devices, such as gloves or suits, assist with motor function recovery for individuals with injuries or neurological conditions [56, 61]. Soft robots are also designed to mimic human organs, like helping the heart pump [62, 63] or aiding bladder function [58]. These applications show how soft robotics is transforming healthcare by making it safer, more effective, and more comfortable for patients.

Table 1 presents a range of emerging applications of soft robotics in the medical field, detailing key systems, their primary functionalities, and current approval status. These examples illustrate the growing impact of soft robotic technologies across diverse biomedical domains, including minimally invasive surgery, rehabilitation, drug delivery, and organ support, highlighting their versatility and potential to transform conventional medical procedures. Despite their promise, relatively few soft robotic devices have received clinical approval to date. Several barriers contribute to this gap between research and clinical translation. These include (1) challenges in precise control of motion, (2) limited durability of soft materials under repeated use, and (3) difficulties in integrating sensors and power sources into flexible structures. Additionally, (4) the translation of soft robotic devices from laboratory prototypes to clinically approved tools requires rigorous testing for biocompatibility, safety, and performance.

**TABLE 1:** Representative applications of soft robotics in the medical field.

Systems	Functionalities	Approval state	Refs.
***Flexible soft robots for MIS/MIB***			
Ferromagnetic soft robot for MIB	Magnetic soft catheter for in situ bioprinting; demonstrated hydrogel printing on organ surfaces.	Research phase	[59]
Bioinspired soft robot system for MIS (Figure 2A)	Hydraulic soft robot with leech‐inspired grasper for high‐force tissue manipulation in Endoscopic Submucosal Dissection	Research phase	[52]
F3DB—Flexible 3D bioprinter for MIB (Figure 2B)	Soft robotic arm delivers multilayer biomaterials in situ; demonstrated in artificial colon and pig kidneys.	Research phase	[10]
Ferromagnetic soft continuum robots (Figure 2C)	Submillimeter‐scale magnetic steering with hydrogel skin for low‐friction access to cerebral aneurysms and laser treatment of vascular stenosis	Research phase	[53]
***Drug delivery***			
MANiACs	Soft microrobots for targeted drug delivery in neural tissues; controlled via magnetic fields	Research phase	[60]
MagCaps (Figure 2D)	Magnetic soft capsule for GI drug delivery; supports dual‐drug release and therapy triggering	Research phase	[54]
***Inner curing***			
Micro‐Fiberbots	Steerable magnetic microbots for inner curing in neurovascular systems, enabling occlusion and shape morphing	Research phase	[64]
Magnetic multi‐layer soft robot (Figure 2E)	Multi‐layer soft robot for wet tissue adhesion and controlled release using magnetic actuation	Research phase	[55]
***Rehabilitation and assistance***			
Soft robotic glove (Figure 2F)	Wearable glove assists hand movement for at‐home rehabilitation using hydraulic actuators.	Research phase	[56]
Soft hip exosuit	Personalized gait support using soft actuators and adaptive control to reduce walking effort	Research phase	[61]
Parkinson's hip assist device	Prevents freezing of gait using cable‐driven hip actuators in a wearable garment	Research phase	[65]
***Prostheses and artificial organs***			
Soft robotic hand (Figure 2G)	Anthropomorphic soft robotic hand mimicking human‐like dexterity for complex object manipulation.	Research phase	[57]
Heart sleeve	Soft sleeve supports cardiac function by synchronizing mechanical compression with heartbeats.	Research phase	[62]
Soft robotics bladder (Figure 2H)	An implantable soft robot assists bladder function via magnetic actuation and tissue compression.	Research phase	[58]

Despite these obstacles, rapid advancements in smart stimuli‐responsive materials, embedded sensing technologies, adaptive control algorithms, and advanced manufacturing techniques are steadily improving the performance and reliability of soft robotic systems [67, 68].

Returning to the context of bioprinting, conventional methods typically rely on bulky, rigid gantry systems that are primarily restricted to ex vivo applications. Conversely, realizing the potential of in situ bioprinting necessitates catheter‐based delivery platforms, for which soft robotics emerges as the premier candidate. Extensive research has demonstrated the efficacy of soft robotic catheters in MIS; for instance, the STIFF‐FLOP system [69, 70] represents a pioneering concept in this domain.

Recent advancements in MIB further underscore the immense potential of soft robotics for internal organ repair. As highlighted in Section 1, the distinguishing advantage of soft robotics over rigid counterparts is mechanical compliance [3, 47]. This property is paramount when navigating natural orifices, as it mitigates the risk of trauma to delicate tissues. Unlike rigid instruments, a soft catheter compliantly deforms upon collision with intestinal walls or internal organs, thereby preserving the integrity of fragile structures such as blood vessels, mucosal linings, and the heart. As detailed in the first section of Table 1, ‘Flexible soft robots for MIS/MIB’ exemplifies the deployment of soft robotics in minimally invasive procedures. Structurally, MIS and MIB systems share a common soft catheter foundation, differing primarily in their end‐effectors. While surgical robots may employ graspers for resection in the ESD method (Figure 2A) or laser treatment of vascular stenosis (Figure 2C), MIB systems integrate specialized printheads and microfluidic channels to deposit bioinks, as shown in Figure 2B.

Taken together, these advances indicate that soft robotics provides the compliant, steerable, and tissue‐safe manipulation and delivery capabilities required for minimally invasive access. Consequently, soft robotics serves as the essential mechanical foundation for MIS in general and MIB in particular, effectively transforming bioprinting from an ex vivo fabrication method into a minimally invasive in situ surgical tool. In the next section, we build on this foundation and review how these same soft‐robotic principles enable MIB, where flexible robotic platforms navigate to internal defects and precisely position printheads for in situ bioink deposition.

## 3 Bioprinting Technologies and Soft Robotic Platforms for In Situ Applications

### 3.1 Comprehensive Overview of 3D Bioprinting

3D bioprinting is an additive manufacturing technique that utilizes Computer‐Aided Design (CAD) to fabricate anatomically precise, functional structures from biomaterials by deposition of living cells for tissue repair and organ regeneration [71, 72].

**FIGURE 3:**
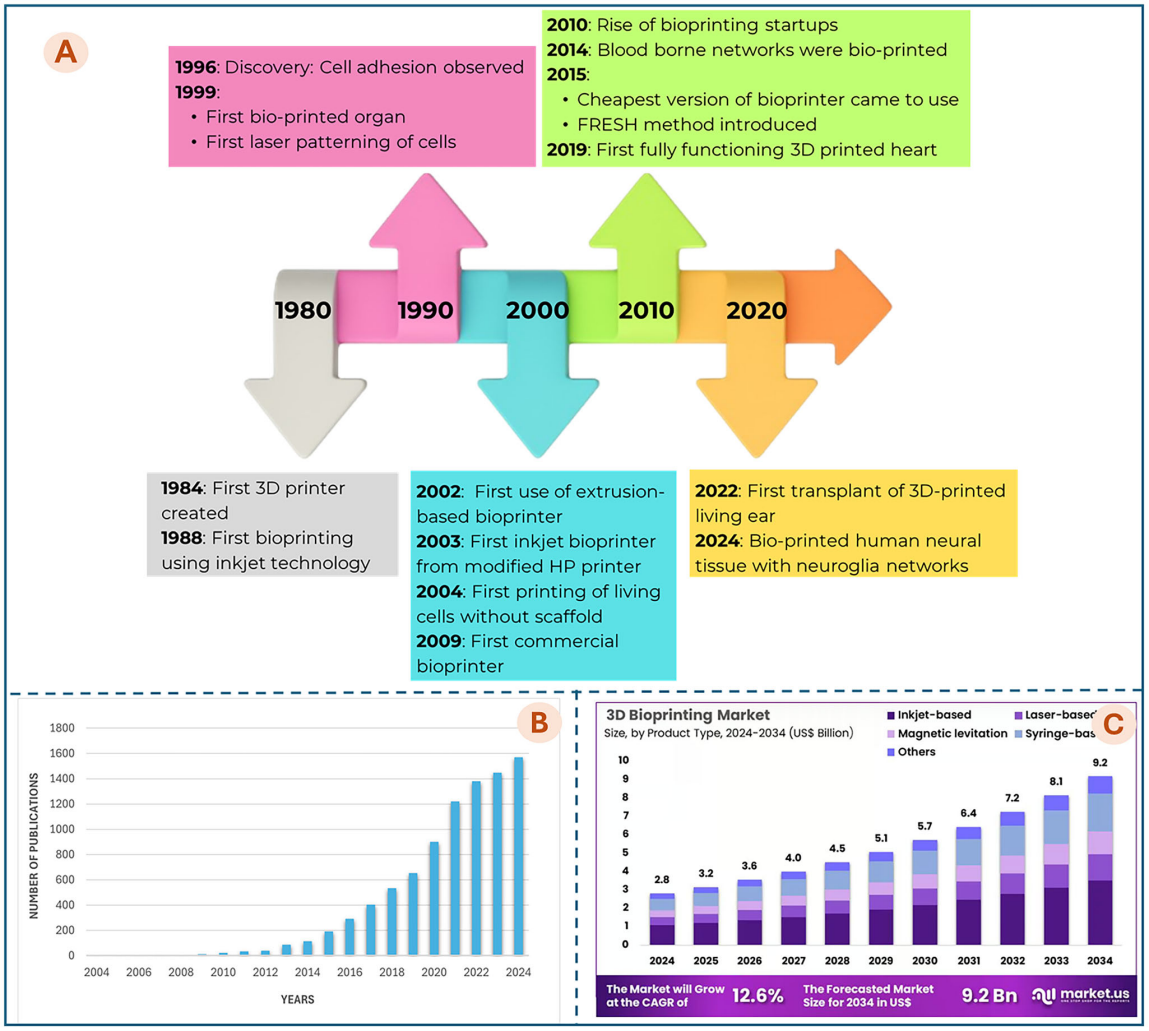
A) Timeline of major milestones in bioprinting development. (B) Trends in 3D bioprinting research publications (Data analysis was searched in the Scopus database in April 2025). (C) Global market forecast for 3D bioprinting technologies from 2022 to 2034 (data collected from Market.us) [66].

3D bioprinting is an additive manufacturing technique involving the printing of living cells and biomaterials at pre‐defined positions based on Computer‐Aided Design (CAD) models to fabricate multi‐layered 3D structures that are anatomically precise and functional for tissue repair and organ regeneration [71, 72]. Figure 3A presents a timeline of the key milestones in the development of bioprinting technology. Since the 2000s, publications on bioprinting have gradually increased, and bioprinting began to receive noticeable research attention around 2012 (Figure 3B). Since then, interest has grown steadily, with a significant acceleration starting from 2018. This trend reflects the rapid expansion of the field and its increasing importance in biomedical research and tissue engineering. ​The global 3D bioprinting market is experiencing significant growth, projected to expand from USD 1.6 billion in 2022 to USD 6.9 billion by 2032, reflecting a compound annual growth rate (CAGR) of 16.1% as shown in Figure 3C. This growing number of scientific publications and the rapidly expanding market clearly show that 3D bioprinting is gaining both academic and commercial attention. It is no longer just an experimental idea, but a promising technology with real‐world medical applications. As research continues to improve the printing of living tissues, with better precision, stronger biomaterials, and higher cell survival, bioprinting is expected to play an essential role in future healthcare [4, 73].

The origins of bioprinting can be traced back to 1984, when Charles Hull invented stereolithography (SLA), marking the inception of 3D printing [74]. Just a few years later, in 1988, Robert J. Klebe pioneered the first demonstration of bioprinting by using a standard Hewlett–Packard inkjet printer to deposit cells through a method known as cytoscribing [75]. In 1996, Forgacs and colleagues provided a foundational understanding of tissue cohesion by relating it to molecular adhesion between cells, introducing the concept of apparent tissue surface tension [76]. By 1999, advancements included the application of laser‐assisted bioprinting by Odde and Renn to position living cells [77], and the Wake Forest Institute, led by Prof. Anthony Atala, successfully engineered the first synthetic human bladder using a patient's own cells, an implant that sustained normal life for over a decade [78]. In 2002, extrusion‐based bioprinting was introduced by Landers et al., later commercialized as the 3D‐Bioplotter [79]. A year later, in 2003, Boland and Wilson adapted a desktop inkjet printer for bioprinting research with biological materials [80]. In 2004, Jakab and his team developed a scaffold‐free bioprinting technique, promoting direct biodegradation [81]. In 2007, the in situ bioprinting technique was first proposed by Weiss in the tissue engineering field, which involved the use of inkjet bioprinting technology [82], followed by Organovo, which partnered with Invetech (Australia) to launch a new bioprinting system calledNovo Gen MMX Bioprinter in 2009. Since 2010, numerous bioprinting startups have emerged, driving innovation in tissue engineering. Notable companies include Organovo, Cyfuse Biomedical, Aspect Biosystems, and BioBots (now Allevi). In 2015, the INKREDIBLE bioprinter was released at about $5000, greatly improving lab accessibility. That year, Carnegie Mellon's Feinberg lab introduced the FRESH (Freeform Reversible Embedding of Suspended Hydrogels) method, enabling high‐resolution printing of soft materials within a gelatin support bath [83]. Breakthroughs continued in 2019, when Noor et al. successfully printed a perfusable miniature heart [84], followed closely by Lee et al., who used FRESH to bioprint collagen‐based human heart structures at multiple scales [85]. In 2020, Feinberg's lab created a full‐size heart model with FRESH printing for surgical planning, using alginate without living cells [86]. That same year, 3DBio Therapeutics successfully fabricated and implanted a 3D‐printed ear made from a patient's own living cells, marking a breakthrough in regenerative medicine. In 2024, Su‐Chun Zhang's team at UW–Madison developed a 3D bioprinting method to fabricate human brain tissues where neurons and astrocytes form functional networks and synapses within and across layers [68]. More recently, CytexOrtho (a U.S company) received FDA clearance to begin its first human clinical trials for the ReNew Hip Implant, which uses bioprinted cartilage to repair hip joints [87].

**FIGURE 4:**
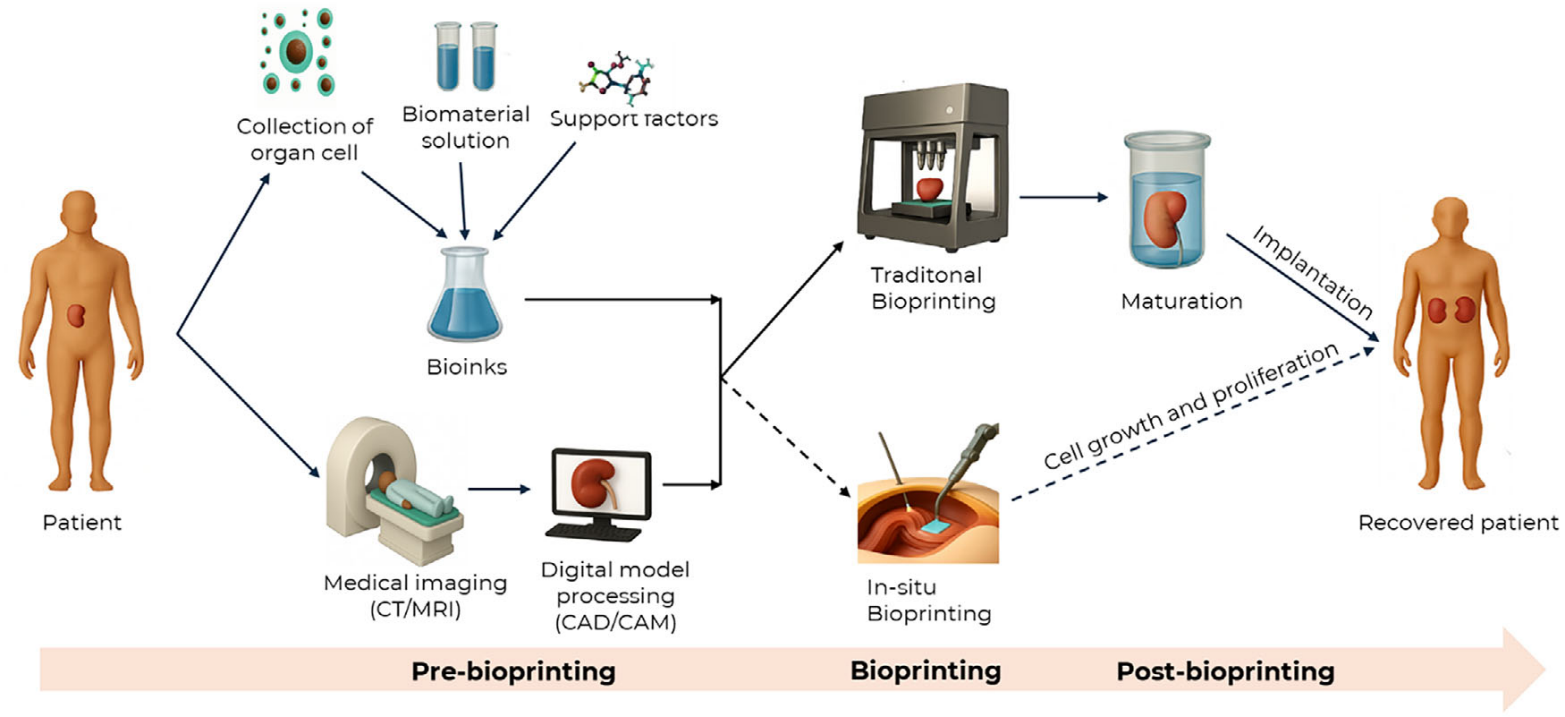
Schematic representation of the bioprinting process.

The general workflow of 3D bioprinting, as shown in Figure 4, consists of three main phases: pre‐bioprinting, bioprinting, and post‐bioprinting [4, 88]. In the pre‐bioprinting stage, cells are harvested and mixed with biomaterials to create bioinks, and 3D models of target tissues are generated from imaging data such as Magnetic Resonance Imaging (MRI) or Computed Tomography (CT). This model is then used to guide the bioprinter during the printing stage, where bioinks are deposited layer by layer to form a living structure with high spatial precision. Finally, post‐bioprinting involves incubating the printed construct for tissue maturation and functionality assessment. An advanced and cutting‐edge approach known as in situ bioprinting allows bioinks to be printed directly onto the patient's body, bypassing the need for post‐printing incubation [9, 33]. This emerging technique represents a significant leap toward real‐time, personalized tissue repair and will be explored in greater detail in a later section.

Bioprinting technologies are rapidly evolving and can be broadly categorized into three primary modalities: extrusion‐based [89, 90], jetting‐based [91, 92], and vat photopolymerization‐based bioprinting [93, 94]. The choice of a suitable bioprinting technique highly depends on the intended application, bioink properties, unique advantages, and limitations of each bioprinting technique. To identify the most suitable bioprinting approach, researchers must weigh the intended clinical use and bioink specifications against the distinct performance profiles and drawbacks of each modality.

Extrusion‐based bioprinting (EBB) functions by dispensing bioinks through a nozzle under controlled pressure to generate continuous filaments, constructing 3D structures layer‐by‐layer. These systems are primarily classified into pneumatic or mechanical (piston or screw‐driven) configurations depending on the dispensing mechanism. EBB is highly valued for its material versatility, capable of processing high‐viscosity bioinks and supporting physiologically relevant high cell densities, including cell spheroids. However, its utility is constrained by shear stress‐induced cell damage at the nozzle tip and a relatively lower resolution (typically >100 µm) compared to light‐based modalities [89, 90].

Jetting‐based bioprinting utilizes a non‐contact, drop‐on‐demand (DOD) mechanism to deposit bioinks as discrete droplets, encompassing diverse techniques such as inkjet (thermal and piezoelectric) [95], electrohydrodynamic (EHD) jetting [96], laser‐induced forward transfer (LIFT) [97], acoustic [98], and microvalve bioprinting [99]. The non‐contact nature of these processes minimizes direct mechanical contact with the substrate and facilitates high‐precision cell placement. In particular, specialized EHD jetting is capable of generating droplets significantly smaller than the nozzle diameter (down to the sub‐micron scale), offering superior precision [91]. However, jetting‐based bioprinting is constrained by stringent bioink rheological and printability requirements, which often necessitate low‐viscosity formulations in several jetting modalities. As a result, the printed constructs typically exhibit limited shape fidelity and weak mechanical integrity, thereby restricting the fabrication of large, load‐bearing 3D tissue constructs [91, 100].

Vat photopolymerization‐based bioprinting constructs 3D tissues by selectively solidifying liquid photosensitive bioinks layer‐by‐layer using light irradiation. This category primarily includes Stereolithography (SLA) [101], which uses a scanning laser beam, Digital Light Processing (DLP) [102], which projects an entire layer image simultaneously using micromirrors, and Two‐Photon Polymerization (TPP) [103] for sub‐micron fabrication. A distinct advantage of this nozzle‐free modality is its superior structural fidelity and resolution (ranging from tens of micrometers in DLP to <100 nm in TPP), eliminating shear stress‐induced cell damage common in extrusion systems. However, its clinical application faces challenges regarding the potential cytotoxicity of photoinitiators and UV light exposure, necessitating the development of biocompatible visible‐light‐curable bioinks [94].

Each of these methods plays a crucial role in advancing organoid research and regenerative medicine by offering diverse strategies to recreate complex, functional biological structures. A wide range of internal organs, including the heart [84], liver [104], kidney [105, 106], lung [107, 108], skin [109], cartilage [110], bone [111], blood vessel [112] and intestine [113], have become active targets of bioprinting research, with many studies making significant strides in developing organ‐specific bioinks, vascular structures, tissue‐mimetic scaffolds, and printing strategies tailored to the unique structural and functional demands of each organ.

The clinical scalability of in situ biofabrication is primarily dictated by the target tissue type, with wound dimensions ranging from localized millimeter‐scale cartilage defects to extensive centimeter‐scale skin injuries. For surface‐level applications such as skin regeneration, handheld bioprinters offer a unique advantage in clinical scalability by enabling the coverage of large‐scale defects in a single continuous pass, regardless of the total area. These centimeter‐scale interventions have been successfully demonstrated on full‐thickness porcine skin wounds as large as 2 cm × 4 cm [114]. Furthermore, research by Albanna et al. has documented repairs for extensive defects reaching 10 cm × 10 cm [115], while other studies in porcine models have addressed circular wounds with a diameter of 2.54 cm (1 inch) [116]. In contrast, cartilage defects, specifically chondral or osteochondral lesions, are generally characterized by smaller, millimeter‐scale areas localized at the surfaces of joint condyles. These interventions often target precisely defined geometries; for example, cylindrical osteochondral defects in rabbit models have been repaired using 5 mm diameter constructs with a depth of 4 mm [15]. Studies in larger animal models have further explored the repair of 8 mm diameter cylindrical defect sites on the weight‐bearing surfaces of ovine femoral condyles [117], while larger circular chondral defects reaching 16 mm in diameter and 4 mm in depth have been treated on bovine femurs [118].

For internal organ repair, MIB platforms are primarily designed to address pathological lesions such as gastric and esophageal ulcers. A paper reported that chronic gastric ulcer size was quantified as ulcer area, with a mean of 49.0 mm^2^ (corresponding to an equivalent circular diameter of approximately 7.9 mm) and a range from 4 to 2960 mm^2^ [119]. Esophageal ulcer size was estimated endoscopically by comparing the ulcer to the known span of an opened biopsy forceps, and was ∼2.8 cm on average [120]. Another significant application for in situ bioprinting involves the repair of iatrogenic lesions resulting from Endoscopic Submucosal Dissection (ESD). During the ESD procedure, a circumferential incision is performed around a gastrointestinal tumour to ensure complete resection [121]. Tumor size was reported as an equivalent circular diameter, ranging from 3.5 to 6 cm, with a mean of 4.6 cm [122]. As a result, the bioprinting system must be capable of treating defect sites that are slightly larger than the original lesion diameter; for a 5 mm polyp, this typically translates to a 6 to 7 mm surgical wound following circumferential resection.

Despite major advances, no in situ bioprinted product with living cells has FDA approval yet, mainly due to regulatory hurdles and the early stage of these technologies. Several FDA‐cleared devices use 3D printing with biocompatible or bioresorbable materials – such as implants and scaffolds – but these are not considered “true bioprinting” because they do not incorporate living cells during fabrication. For example, although the OsteoFab cranial implant [123] and the 3DMatrix surgical mesh [124] were both 3D‐printed and approved to facilitate tissue regeneration or support healing, they do not feature cell‐laden bioinks. Similarly, the ReNew Hip Implant, developed by CytexOrtho, uses a special printed structure designed to mimic cartilage and has been approved for clinical trials [87]. However, it remains in its early stages of development and does not yet enable the direct printing of living cells. While not yet constituting “true bioprinting” due to the absence of living cells, these advanced technologies demonstrate the transformative potential of 3D printing in medicine by supporting tissue repair and regeneration. They mark important milestones toward the development of fully functional, cell‐laden implants that could eventually repair or replace entire organs.

Looking ahead, integrating bioprinting with 4D bioprinting, organ‐on‐chip systems, AI, and machine learning (ML) is expected to accelerate progress toward fully functional, patient‐specific organs. These advances could address the global organ shortage and transform the treatment of damaged or diseased tissues. Section 5 provides a detailed analysis of these directions.

### 3.2 Soft Robotic Approaches for In Situ Bioprinting

As in situ bioprinting moves toward clinical translation, devices that deposit cell‐laden bioinks safely, precisely, and with minimal tissue trauma are essential. Traditionally, bioprinted constructs are either matured in vitro before implantation or fabricated externally and implanted via open surgery [33, 72, 127, 128, 129]. Over the last few decades, these procedures have relied heavily on large‐form‐factor 3D bioprinters, both commercial and research‐grade [71] such as the NovoGen MMX Bioprinters (Organovo, Delaware, USA), 3D Discovery (RegenHU, Switzerland), INKREDIBLE (Cellink, Switzerland), BIOBOT TM and BIOASSEMBLYBOT (Advanced Solutions, USA), BIO3D (Singapore), and the RASTRUM 3D (Inventia Life Science, Australia) [89]. While in vitro bioprinting has been instrumental in the early stages of tissue engineering, it presents several critical limitations that hinder clinical translation. (1) The bioprinted constructs often exhibit poor initial mechanical strength due to hydrogen‐based bioinks, rendering them fragile and difficult to handle during implantation [130]. For example, physical fixation of in vitro constructs to native tissues, such as suturing or pressing, can damage the micro‐ and macro‐architecture of the printed structure, adversely affecting cell viability and tissue integration. (2) There is frequently a morphological mismatch between prefabricated constructs and the actual defect site, necessitating additional intraoperative trimming or adjustments that prolong surgical time and compromise mechanical integration [32]. (3) The workflow from printing to implantation involves multiple steps, fabrication, culture, storage, and transport, all of which must adhere to strict sterility protocols, increasing the risk of contamination [33]. (4) The separation between fabrication and biological maturation stages results in limited physiological conditioning of the constructs, which can impair post‐implantation performance. (5) Finally, manual implantation procedures introduce variability and lower tolerance to procedural errors, making in vitro approaches less attractive for time‐sensitive or precision‐demanding clinical interventions.

In situ bioprinting has emerged as a promising alternative to overcome the limitations of traditional in vitro methods, offering several potential advantages as outlined below. (1) It may overcome the mechanical fragility and transport issues of prefabricated constructs by enabling direct deposition of bioinks inside the body [33]. (2) This strategy possesses a great potential to allow printing to be tailored in real‐time to the defect site, significantly improving geometric fidelity and reducing surgical complexity [30]. (3) In situ bioprinting can eliminate the need for scaffold transport and implantation, reducing risks of contamination and mechanical damage [33]. (4) By utilizing the patient's body as a biological environment from the start, in situ bioprinting potentially fosters immediate tissue–host interaction and more effective integration [72]. (5) Finally, it aligns with the trend toward minimally invasive, robot‐assisted procedures, offering improved precision and reduced patient trauma, as conventional approaches often involve large, invasive incisions to implant printed scaffolds or organs [9]. In situ bioprinting holds significant promise for personalized and intraoperative regenerative therapies, though the field remains in its infancy, with current applications limited to skin, bone, and cartilage repair and no clinically approved bioprinters to date, underscoring the need for advances in device design, miniaturization, regulation, and translational research [33].

**FIGURE 5:**
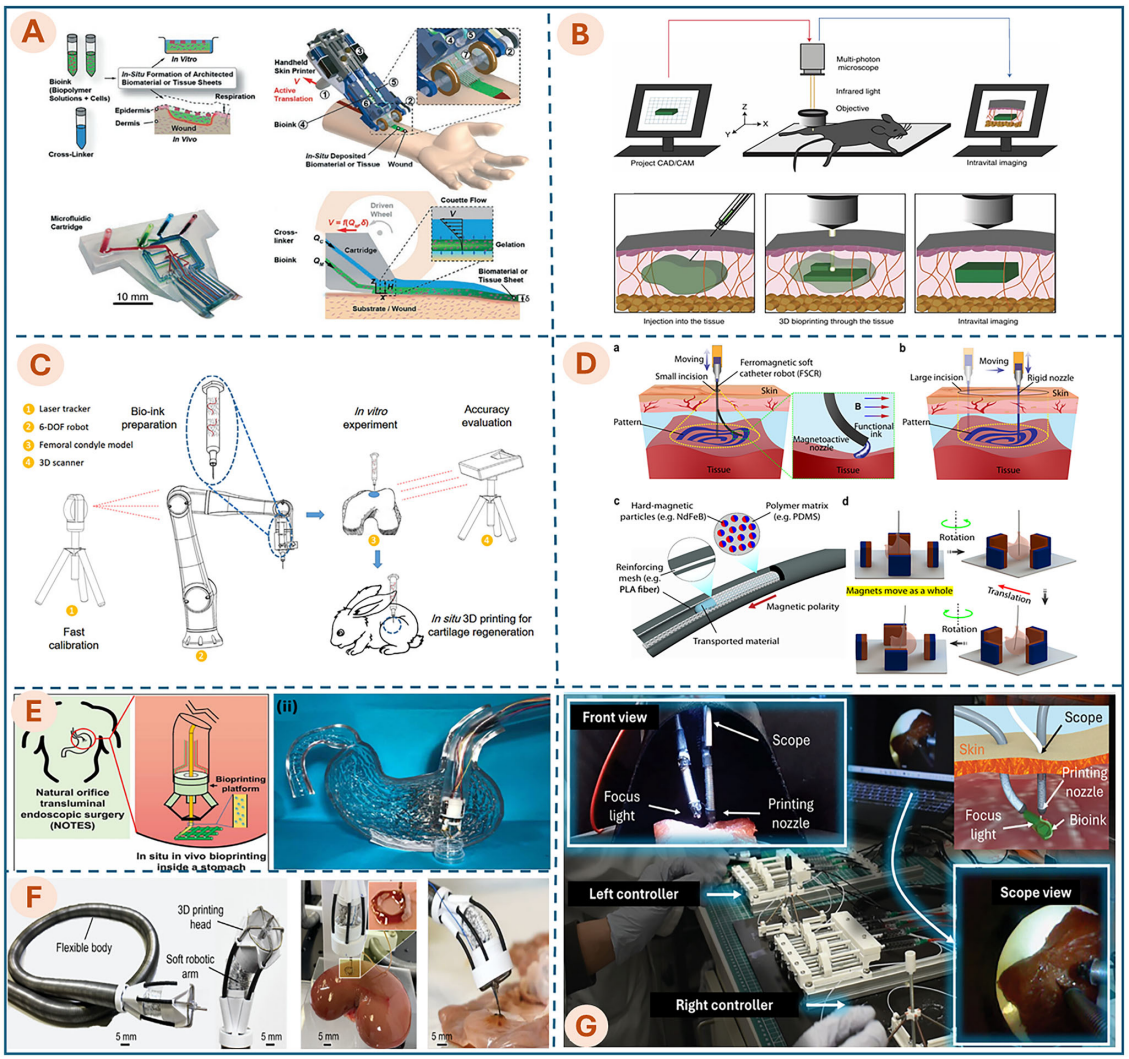
Representative promising platforms for 3D in situ biofabrication and bioprinting techniques. Illustrative examples of state‐of‐the‐art in situ bioprinting systems: (A) Handheld Skin Printer. Reproduced with permission from ref. [125] Copyright 2019, John Wiley and Sons. (B) Near‐infrared polymerization bioprinting system. Reproduced with permission from ref. [126]. Copyright 2023, SAGE Publications. (C) Industrial robot (6‐DOF) system. Reproduced with permission from ref. [15]. Copyright 2020, Elsevier. (D) Ferromagnetic soft catheter robot (FSCR). Reproduced with permission from ref. [59]. Copyright 2021, Springer Nature. (E) Microbioprinting platform. Reproduced with permission from ref. [18]. Copyright 2022, Cell Press. (F) Flexible in situ 3D bioprinter (F3DB). Reproduced with permission from ref. [10]. Copyright 2023, John Wiley and Sons. (G) Motor‐free soft robotic system [23]. Copyright 2025, John Wiley and Sons.

While existing reviews of in situ bioprinting predominantly focus on handheld devices and collaborative robotic platforms [5, 30, 33], the present review broadens this classification to include flexible soft robotic systems, as they provide a distinct and clinically relevant pathway for minimally invasive access to deep‐seated anatomical sites, including delivery through natural orifices and confined lumens. Under the conventional definition of three‐dimensional bioprinting, which typically implies a CAD‐ or imaging‐guided, patient‐specific workflow and the deposition of living cells within biomaterials, many current handheld and flexible platforms should be regarded as early‐stage or partially integrated systems. A recurring limitation is the lack of patient‐specific defect acquisition, such as intraoperative scanning or imaging, from which individualized toolpaths can be generated. For instance, Hakimi et al. demonstrated multilayer deposition of cell‐laden bioinks; however, the operation remains largely manual and is not guided by defect‐site imaging (Figure 5A). More recently, Wang et al. presented a handheld platform capable of programmable trajectory execution [14], but it has not yet been validated with cell‐laden bioinks, thus representing an incomplete in situ bioprinting workflow. Similarly, most flexible robotic systems (e.g., Figure 5D,F) rely on pre‐programmed trajectories of simple geometries (e.g., squares or circles) rather than reconstructed paths tailored to actual wound morphologies. Nevertheless, these platforms can be discussed as promising in situ bioprinting devices because they already deliver essential hardware capabilities for the in situ deposition phase, including controlled extrusion and multilayer patterning. As patient‐specific sensing and reconstruction, along with automated planning and control, continue to mature, these systems are expected to evolve toward a fully integrated in situ bioprinting pipeline.

To facilitate a systematic comparison, the subsequent paragraphs discuss each device category in turn. Table 2 compares key characteristics across these categories, including platform configuration, printing modality, experimental models, and target tissues, thereby highlighting their respective strengths and limitations. Table 3 further evaluates control strategies, cell viability, printing resolution, and overall accuracy, providing a structured basis for assessing the potential of soft robotic technologies to advance MIB. Figure 5 illustrates representative state‐of‐the‐art platforms discussed in this section.

**TABLE 2:** Representative in situ bioprinting devices and their key characteristics.

Device	Description	Platform type	Printing modality	Potential target tissue	Experimental model	Refs.
Handheld Skin Printer (Figure 5A)	A portable extrusion device for in situ skin regeneration enables rapid, multilayer deposition of cell‐laden bioink directly onto wounds.	Handheld	Extrusion‐based	Skin wounds (epidermis, dermis)	A murine and porcine wound model (ex vivo)	[114]
Intravital 3D Bioprinting (Figure 5B)	A non‐contact laser‐based bioprinting system for direct in vivo tissue construction, forming complex structures inside tissues using two‐photon activation.	Multiphoton microscope with motorized XYZ stage	Laser‐assisted photopolymerization	Anatomical sites	The dermis, skeletal muscle, and brain of live mice (in vivo)	[131]
6‐DOF industrial robotic arm (Figure 5C)	An industrial robotic arm system for cartilage/bone defect repair; it performs precise in situ scaffold deposition and enhances tissue regeneration in vivo.	Industrial robot arm	Extrusion‐based	Articular cartilage	Knee joint defect of a rabbit (in vivo)	[15]
Ferromagnetic Soft Catheter Robot (Figure 5D)	A magnetically controlled soft catheter for minimally invasive in situ gel delivery; prints patterns on curved internal surfaces with remote magnetic guidance.	Flexible soft robot	Extrusion‐based	Internal organ	Liver of rat (in vivo); porcine stomach phantom (ex vivo)	[59]
Micromachine printer (Figure 5E)	A miniaturized robotic system for endoscopic bioprinting, capable of precise deposition of layered bioink in gastric environments through rigid shafts.	A micro delta mechanism is built into an endoscopic shaft	Extrusion‐based	Gastric mucosa	Porcine stomach phantom surface (ex vivo)	[10]
F3DB soft robotics system (Figure 5F)	A soft robotic arm for bioprinting inside body cavities; it navigates confined anatomy, delivers multilayer hydrogel, and supports surgical tasks.	Flexible soft robot	Extrusion‐based	Internal organ	Colon, bladder, and kidney of a pig (ex vivo)	[10]
Motor‐free soft robotic system (Figure 5G)	A portable, motor‐free soft robotic platform, designed for conducting MIS within complex internal organs	Flexible soft robot	Extrusion‐based	Internal organ	Porcine tissue (ex vivo)	[23]

**TABLE 3:** Comparative analysis of control complexity, feedback, and bioprinting performance in emerging in situ bioprinting platforms.

Device	Control techniques	Feedback mechanism for a control system	MIB	Bioinks/ Biomaterials	Cell Viability	Printing resolution	Printing accuracy	Refs.
**Handheld devices**								
Handheld skin printer (Figure 5A)	Manual handheld guidance. Integrated rollers and a microfluidic cartridge coordinate the operator's translation speed with the bioink flow rate.	Human‐in‐the‐Loop (via visual feedback)	**No** (Stage 1)	Polysaccharide‐based bioink (Alginate + Collagen type I); Protein‐based bioink (Fibrinogen + Hyaluronic acid) and media (DMEM, HEPES, PBS)	>90% (after 10 days)	An analytical model governs nominal resolution	Highly dependent on the operator's manual guidance	[114]
**Collaborative/Rigid robotic system**								
Intravital 3D Bioprinting (Figure 5B)	CAD/CAM‐driven, using a motorized XYZ stage to guide the multiphoton microscope's laser for accurate 3D positioning.	Human‐in‐the‐Loop (via visual feedback)	**Yes** (Stage 3)	Cell‐laden, photosensitive hydrogel of HCCA‐conjugated Gelatin or branched PEG.	90–99% (after 2 days)	Sub‐micrometric resolution; minimal line width: 1.9 ± 0.2 µm	High spatial precision; Accurate positioning and orientation of the bioprinted structures	[131]
6‐DOF robotic arm (Figure 5C)	A robot arm with custom programming software, a fast calibration method using a laser tracker, and kinematic modeling for accuracy	Open‐Loop (no sensory feedback)	**No** (Stage 2)	A cell‐free hydrogel of Hyaluronic Acid Methacrylate (HAMA) with Acrylate‐terminated 4‐armed Polyethylene Glycol as a cross‐linker.	N/A	N/A	Average error of printed surface <0.3 mm Standard deviation (SD): 0.1447 mm	[15]
Micromachine printer (Figure 5E)	A miniaturized Delta robot actuated by three stepping motors, controlled by an inverse kinematics model that generates printing trajectories	Open‐Loop (no sensory feedback)	**Yes** (Stage 4)	Gelatin–alginate hydrogel with human gastric epithelial cells (GES‐1 cells) and human gastric smooth muscle cells (HGSMCs)​	>90% (after 10 days)	Average thread diameter: 0.5 mm	Average deviation: 1.18mm±0.43 mm in a latticed structure (side dimension: 14 mm); 0.67 mm ± 0.15 mm in a circle (radius: 10 mm)	[18]
**Flexible soft robotics system**								
Ferromagnetic Soft Catheter Robot (Figure 5D)	Magnetic actuation via four computer‐controlled permanent magnets, computer‐guided motion via a pre‐calibrated mapping relationship	Open‐Loop (no sensory feedback)	**Yes** (Stage 3)	Various cell‐free functional inks (Ecoflex‐PDMS, conductive silver, hydrogels) were tested to demonstrate system versatility.	N/A	Best reported resolution: 0.53 mm	High accuracy (demonstrated by excellent agreement with digital designs)	[59]
Flexible in situ 3D bioprinter (Figure 5F)	A hydraulic‐driven soft robot controlled by an inverse kinematic model, using model‐based feedforward and machine learning to compensate for nonlinear hysteresis	Open‐Loop (no sensory feedback)	**Yes** (Stage 4)	Gelatin‐based hydrogel (X‐Pure GelDAT) with L929 cells​	High viability	Minimal width of printed line: ∼ 1 mm	Root mean square error (RMSE) <0.3 mm Standard Deviation (SD) <0.148 mm	[10]
Motor‐free soft robotic system (Figure 5G)	A master‐slave teleoperation system based on soft fibrous syringe architecture, controlled manually via a joystick, eliminating the need for motors and control algorithms.	Human‐in‐the‐Loop (via visual feedback)	**Yes** (Stage 3)	A cell‐free gel composite made from cationic polymers, silicone, alcohol, and olive oil	N/A	Dependent on the operator's skill; reported minimal width of printed line: 0.5 mm	Dependent on the operator's skill; reported RMSE <0.3 mm, SD <0.16 mm	[23]

**Handheld devices** are portable and simple to deploy, making them well‐suited for open surgical settings and external tissue applications [33], particularly in emergency scenarios where immediate, on‐site intervention is required. They are generally effective for superficial tissues; however, scalability and reproducibility are often limited by manual operation, which restricts their applicability to internal targets and minimally invasive procedures. A representative example is the handheld skin printer developed by Hakimi et al. [114] (Figure 5A). This lightweight device, which operates in a manner similar to a correction‐tape dispenser, uses a microfluidic cartridge and rollers to conformally deposit bioink sheets onto wounds. Its main advantages include operational simplicity, portability, and clinical practicality, enabling rapid coverage of large and irregular defects at the point of care. Efficacy was supported by uniform in situ formation of biomaterial sheets containing viable cells and improvements in wound‐healing outcomes in in vivo models. The primary trade‐off is low structural resolution, making the platform more suitable for depositing planar layers on external wounds than for fabricating complex microarchitectures within deep tissues.

In contrast to handheld systems, **collaborative and rigid robotic platforms** provide higher stability and positional accuracy, but their size and reliance on rigid instrumentation typically confine their use to in situ bioprinting during open surgery or settings with direct access to the target site. Urciuolo et al. introduced intravital 3D bioprinting (i3D) as a notable example of high‐precision in situ fabrication [131] (Figure 5B). The approach employs two‐photon crosslinking driven by a near‐infrared femtosecond laser to polymerize a photosensitive bioink directly within living tissue. Beyond its high fidelity, achieving sub‐micrometric resolution, the system uniquely integrates real‐time intravital imaging to support accurate anatomical positioning. Its efficacy was demonstrated by high cell viability and de novo formation of vascularized myofibers from printed donor cells. Nevertheless, the approach remains constrained by the inherent limitations of multiphoton microscopy, including limited fabrication depth (typically <2 mm), restricted printable volume, and the requirement for direct optical access to the target site.

Additional rigid robotic implementations further illustrate the trade‐off between accuracy and invasiveness. Ma et al. developed a 6‐DOF industrial robotic arm system for osteochondral defect repair [15] (Figure 5C). The platform extrudes a photo‐crosslinkable hydrogel directly into knee joint defects under real‐time control, enabling precise scaffold placement without manual intervention, and rabbit studies indicated improved cartilage regeneration relative to untreated controls. While the system offers high accuracy and structural stability, its rigid architecture necessitates open surgery and is not readily compatible with minimally invasive workflows.

In an effort to miniaturize rigid robotic printing for endoluminal access, Zhao et al. developed an endoscope‐mountable bioprinting platform based on a Delta‐robot architecture for gastric applications [18] (Figure 5E). The system is actuated by three compact motors and controlled using an inverse‐kinematics model that converts desired printing trajectories into motor commands. The authors demonstrated the printing of a two‐layer, dual‐cell‐type scaffold mimicking the gastric wall within a stomach model, maintaining high cell viability for 10 days. However, the gelatin–alginate bioink used is thermally unstable at physiological temperature, limiting in vivo applicability. Moreover, despite being mounted to an endoscope, the motion is transmitted through a rigid shaft and mechanism, which reduces compliance and may increase the risk of unintended tissue contact or damage upon collision in confined spaces.

To enable minimally invasive bioprinting (Stage 3 and Stage 4) and to overcome the access limitations inherent to rigid platforms, **flexible soft robotic systems** have emerged as a third category. These systems are designed to traverse tortuous anatomical pathways and reach delicate, hard‐to‐access targets, often adopting master–slave teleoperation to steer and orient the distal tip during navigation while preserving intuitive control and procedural accuracy. Within this category, recent platforms can be discussed in terms of two minimally invasive access paradigms: Stage 3, which targets in situ deposition through small incisions or surgical ports, and Stage 4, which emphasizes endoscopic deployment through natural orifices.

Stage 3 platforms have focused on demonstrating feasibility for controlled internal deposition under constrained conditions using incision‐based minimally invasive access. Zhou et al. developed a ferromagnetic soft catheter robot system that exemplifies this direction [59] (Figure 5D). The system employs a soft catheter fabricated from a ferromagnetic composite and is actuated by four computer‐controlled magnets. Motion is governed by a pre‐calibrated open‐loop model that translates three‐dimensional scan data into G‐code for tip control. The platform demonstrated versatility by printing various cell‐free functional inks and performing minimally invasive deposition of a hydrogel pattern onto a rat liver. Despite this feasibility, clinical translation is challenged by the reliance on large external magnetic actuation and associated shielding requirements, which can impose a substantial system footprint, introduce electromagnetic interference with sensitive clinical equipment (e.g., patient monitors and pacemakers), and limit compatibility with MRI environments or other ferromagnetic‐sensitive settings [6]. In a complementary direction, Nguyen et al. proposed a motor‐free master–slave system (mfSRS) based on a soft fibrous syringe architecture [23] (Figure 5G), in which a manual joystick provides one‐to‐one hydraulic control of a soft arm. This electronics‐free architecture can attenuate operator tremor and simplify actuation; however, its full dependence on manual operation remains a major limitation. Printing accuracy and resolution are strongly operator‐dependent because joystick motion directly determines nozzle trajectory. Moreover, the system does not support automated toolpath generation from patient‐specific imaging data (e.g., CT or MRI), restricting its suitability for applications requiring high anatomical fidelity.

Stage 4 platforms emphasize clinically relevant endoscopic access routes, particularly deployment through natural orifices, and aim to support in situ deposition within internal lumens such as the gastrointestinal tract. Thai et al. introduced the F3DB, a flexible in situ 3D bioprinter designed for multilayer biomaterial deposition during endoscopic procedures in internal organs [10] (Figure 5F). The system integrates a soft robotic arm and a printing head actuated by hydraulic soft muscles, enabling navigation and printing in confined spaces via natural‐orifice access. Operation is achieved through a master–slave framework combined with kinematic modeling and machine‐learning‐based algorithms, and feasibility was demonstrated through bioprinting on ex vivo tissues. Key strengths include its scalability and multifunctionality for endoscopic intervention; however, a limited bending range and the absence of real‐time sensing constrain positioning precision and repeatability during deposition.

As summarized in Table 3, an analysis of the emerging platforms for MIB reveals a clear predominance of soft robotic systems, underscoring the perceived potential of this technology for the field. However, this analysis also reveals significant limitations in their control architecture. Current systems predominantly rely on open‐loop or human‐in‐the‐loop (via visual feedback) control mechanisms. This general absence of integrated sensors and real‐time, closed‐loop feedback inherently restricts printing performance, such as accuracy and resolution, within the dynamic in vivo environment. Additionally, a deficiency in navigation, orientation sensing, and tracking systems restricts the deployment of these tools through natural orifices, thereby limiting access to complex or deep‐seated defect sites (detailed in Section 4.4). Consequently, as the field is still in its nascent stages, current research efforts are largely focused on validating fundamental feasibility by printing simple, pre‐defined geometries (such as circles or rectangles) with a limited number of layers. Furthermore, these systems generally do not yet integrate the capability to automatically generate printing trajectories by scanning patient‐specific defects, a process which is fundamental to in situ bioprinting (discussed further in Section 4.1).

Despite these early‐stage challenges, the potential of soft robotics in revolutionizing internal organ repair is clear, aligning with trends toward less invasive, more personalized, and highly adaptable bioprinting solutions. Realizing this potential hinges on overcoming the specific limitations outlined above. Future development will require advancements in soft actuation to create dexterous, flexible arms and catheters. Concurrently, embedded sensing must be integrated to enable the missing closed‐loop feedback, while imaging technologies are essential for processing pre‐operative patient data to generate automated trajectories. In addition, the development of advanced bioinks remains a cornerstone for ensuring successful tissue regeneration and clinical outcomes. Furthermore, the integration of ML and AI will play a pivotal role in improving control algorithms, enhancing precision, and enabling real‐time adaptation during procedures (discussed more in Sections 4.6 and 5.4). The following sections will, therefore, delve into these critical underpinning technologies and explore the emerging trends expected to propel this field forward.

### 3.3 Trade‐Offs Between Rigid Robotic and Flexible Soft Robotics Platforms for In Situ Bioprinting

The key trade‐offs between rigid and flexible platforms across these dimensions are summarized in Table 4. Rigid robotic arms are well‐suited to Stage 1 and Stage 2 in situ bioprinting because their high structural stiffness provides superior positional accuracy, repeatability, and printing stability. The nozzle pose is less affected by external disturbances or contact forces, which helps maintain consistent extrusion and layer placement. This advantage is particularly important when the target tissue is directly exposed or when the workspace is sufficiently open that the robot can maintain a stable posture without being constrained by access geometry. In Stage 3, where printing is performed through small incisions or surgical ports, rigid robotic arms can still be effective for relatively simple tasks in which the printing surface lies close to the entry point, such as certain demonstrations for cartilage‐related repair. In these cases, the required motion is largely short and straight, and the finite number of joints and degrees of freedom is sufficient to cover the target area without extensive reorientation.

**TABLE 4:** Comparison between rigid robotic arms and flexible soft robots for in situ bioprinting across Stages 1–4.

Characteristic	Collaborative/Rigid robotic arms	Flexible soft robots
Best‐fit stages	Stage 1–2; limited Stage 3 (shallow targets)	Stage 3–4 (deep targets)
Access route	Open surgery/port‐based straight access	Port‐based steering; natural‐orifice/endoscopic
Compliance	Low	High
Safety in crowded anatomy	Lower	Higher
Dexterity	Low	High
Flexibility	Low	High
DOF	Finite	Infinite
Joints	Finite	Infinite
Approach angles at the target	Limited by port pivot	Multi‐angle tip reorientation
Port placement sensitivity	High	Lower (steering compensates)
Multi‐position printing	Limited; may need extra ports	Strong; can treat multiple sites from one entry
Control complexity	Easier	Complex

However, as the target becomes deeper inside the body and the anatomical environment becomes more crowded, rigid shaft‐based instrumentation faces fundamental constraints that directly affect feasibility and safety. When a rigid instrument is inserted through a port, the incision behaves as a fixed pivot, forcing the tool to rotate about that point. This port‐based kinematic constraint produces the fulcrum effect [132], in which the distal tip motion is strongly coupled to the pivot at the entry site, restricting maneuverability and making it difficult to achieve favorable approach angles at the target. As a consequence, the nozzle is harder to align with complex or curved defect geometries, and the operator must rely on careful preoperative port placement because small changes in defect location can substantially affect whether the site can be reached with an appropriate orientation. These limitations become even more significant when multiple defect sites must be treated in a single intervention. If two lesions are located at different regions of an internal organ, such as the stomach, a rigid tool may be unable to reposition the distal tip to the second site while maintaining safe approach angles, and additional ports may be required. Each added port increases procedural trauma and the risk of collateral tissue damage.

Flexible soft robotic systems are specifically attractive in Stage 3 because their high compliance, high flexibility, and high degrees of freedom [51] allow the device to bend and steer within the body after entering through a single access point. Instead of relying on a straight rigid shaft to define the reachable workspace, a flexible robot can deform around anatomical structures and reorient the distal tip to approach a defect from multiple angles. This capability enables sequential printing at separate sites from one incision, improving practicality for distributed defects and reducing invasiveness by avoiding additional ports. For example, when multiple lesions are present on different regions of the stomach wall, a rigid shaft‐based approach may require separate access ports to achieve feasible approach angles to each lesion, whereas a steerable flexible soft robot can print at the first lesion and then navigate internally to the second lesion through the same entry route, without creating an additional incision. Moreover, because steering can compensate for imperfect access geometry, the port placement constraint becomes less strict; entering through a nearby region may still allow the robot to reach the defect with a suitable printing orientation.

These advantages, however, come with important engineering trade‐offs. The same compliance and continuum deformation that provide reachability also introduce hysteresis, nonlinearity, and load‐dependent shape changes, which can reduce open‐loop accuracy and make motion prediction more difficult [47]. As a result, flexible soft robots typically require more sophisticated modeling and control strategies, often relying on closed‐loop compensation, to achieve reliable printing performance and maintain consistent nozzle positioning during deposition on deformable or moving organs.

In Stage 4, where access is achieved through natural orifices and endoscopic routes, the available space is even more constrained, and the access path is typically tortuous. Under these conditions, rigid robotic arms are generally unsuitable because they cannot conform to the access pathway, whereas flexible soft robots are inherently compatible with the navigation and reach requirements due to their high flexibility and steerability. Although current demonstrations remain limited, continued progress in modeling and control is expected to mitigate hysteresis‐related challenges and further strengthen the case for flexible soft robotic platforms in clinically realistic in situ bioprinting.

## 4 Advanced Technologies for Minimally Invasive Bioprinting

**FIGURE 6:**
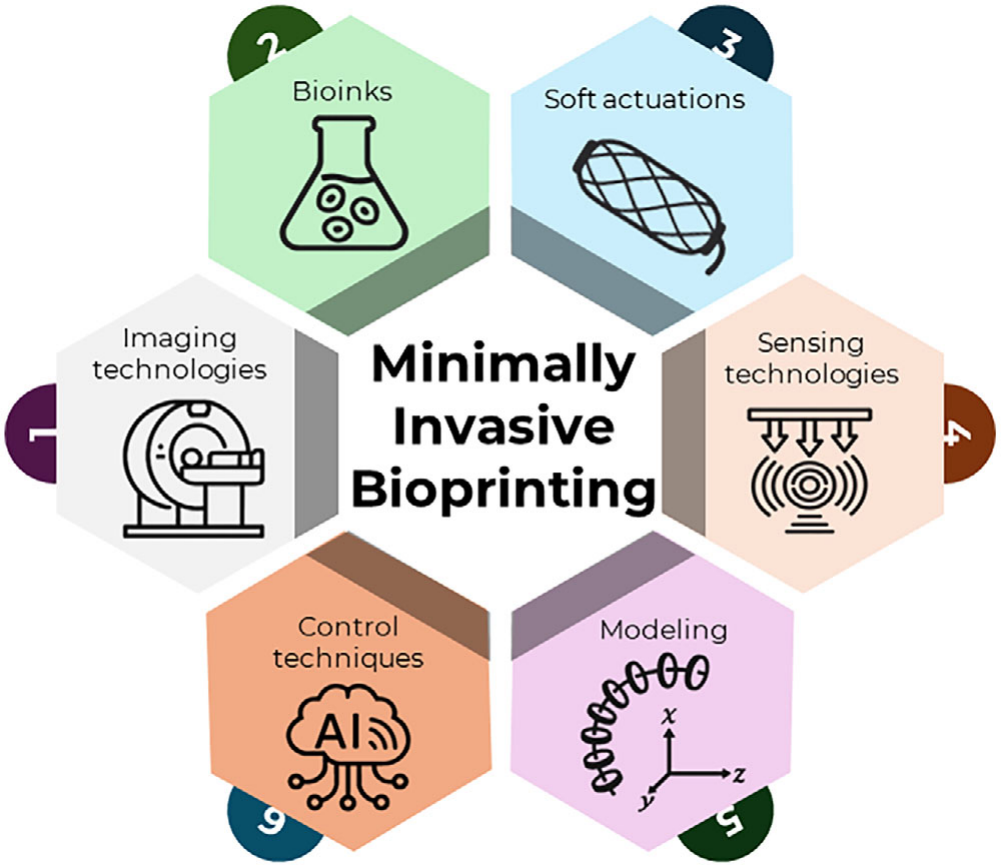
Key enabling technologies for minimally invasive in situ bioprinting. This diagram illustrates six foundational technology pillars essential for the advancement of MIB systems: imaging technologies, bioink preparation strategies, soft actuations, sensing technologies, modeling and control techniques.

MIB requires the coordinated integration of advanced technologies to operate precisely and safely within confined internal environments. Effective platforms must combine imaging and preoperative planning, bioink preparation and crosslinking strategies, and soft, compliant robotic components equipped with real‐time sensing, supported by modeling and control methods for accurate navigation, stable targeting, and controlled bioink deposition. As summarized in Figure 6, we highlight six key technology pillars enabling MIB: imaging, bioink/crosslinking, soft actuation, tactile/kinematic sensing, modeling, and control. The following subsections (4.1–4.6) discuss each pillar in turn, summarizing the current state of the art and the remaining technical challenges toward next‐generation MIB systems.

### 4.1 Imaging Technologies

The integration of patient‐specific defect scanning is fundamental to in situ bioprinting, as it allows the system to obtain precise information on the wound's complex topography. This scan data is then used to automatically generate a custom printing trajectory, ensuring the biomaterials are deposited to accurately adapt to the defect site. A wide range of imaging technologies has been developed to support tissue engineering and bioprinting, including MRI, Positron Emission Tomography (PET), Computed Tomography (CT), Confocal Microscopy (CM), Multiphoton/Two‐Photon Microscopy (MPM/TPM), Selective Plane Illumination Microscopy (SPIM), Optical Coherence Tomography (OCT), Optical Projection Tomography (OPT), Laminar Optical Tomography (LOT), and Photoacoustic Tomography/Microscopy (PAT/PAM) [137]. This section provides a detailed overview of these widely applied imaging modalities, such as CT, MRI, OCT, and SLS, highlighting their principles, capabilities, and specific relevance to in situ bioprinting applications.

**FIGURE 7:**
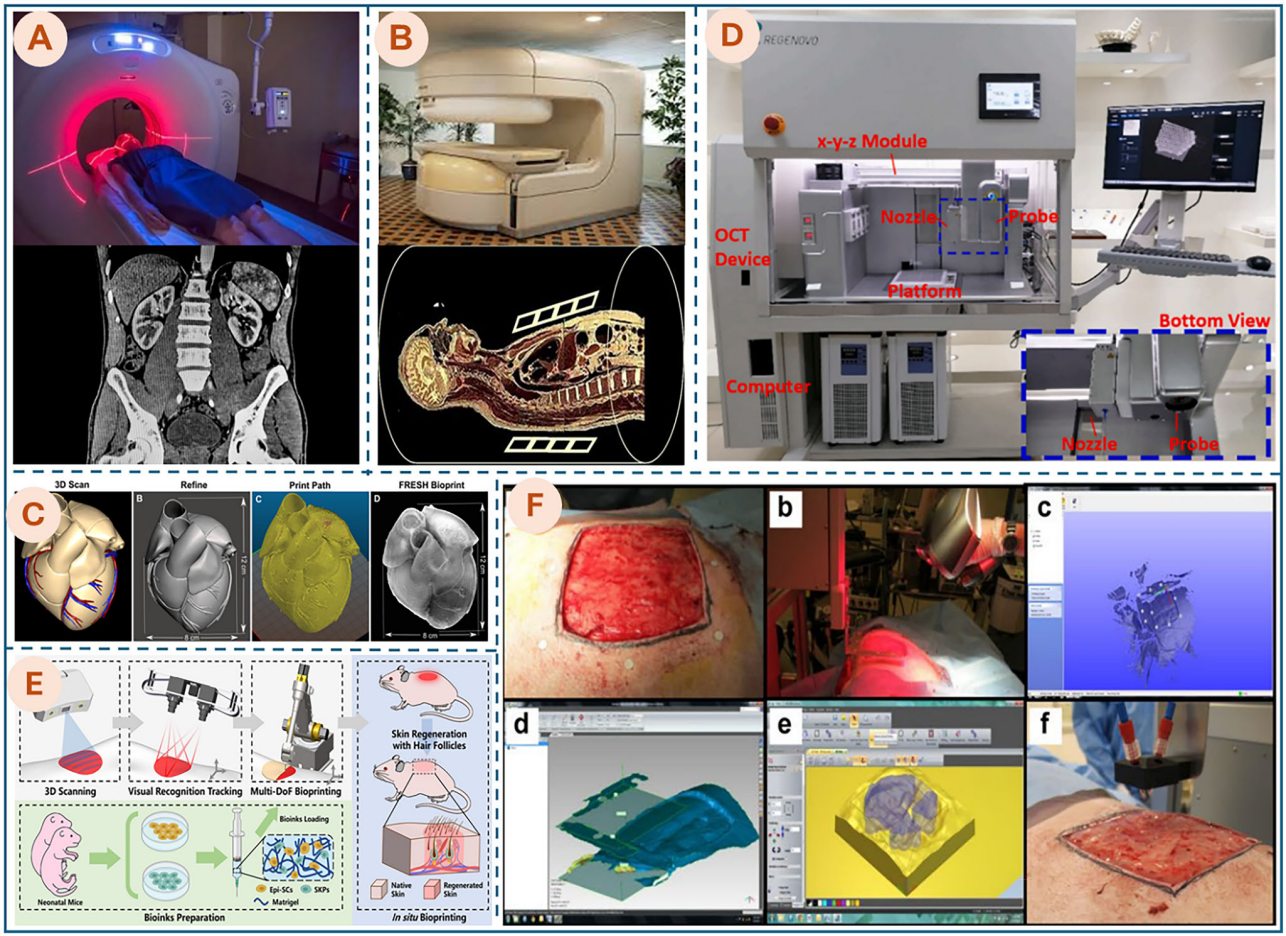
Imaging technology for bioprinting applications. (A) A CT scanner captures cross‐sectional images of the body [133]. Photo courtesy of the National Institute of Biomedical Imaging and Bioengineering. Public Domain. (B) An MRI scanner is used for detailed 3D imaging of internal structures [134]. Photo courtesy of the National Institute of Biomedical Imaging and Bioengineering. Public Domain. (C) The process of creating a 3D printed heart model from MRI data. Adapted with permission from ref. [86]. Copyright 2020, American Chemical Society. (D) An extrusion bioprinter integrated with an OCT system. Adapted with permission from ref. [135]. Copyright 2021, Elsevier. (E) Workflow schematic of the in situ bioprinting robot using structured‐light 3D scanning. Reproduced with permission from ref. [136]. Copyright 2022, Wiley. (F) Overview of the skin bioprinting process using wound markers, 3D scanning, STL modeling, and precise multi‐cell layer printing. Adapted with permission from ref. [115]. Copyright 2019, Springer Nature.

CT utilizes a motorized X‐ray source and digital detectors to capture cross‐sectional images of the body, which are then computer‐processed into tomographic slices. These slices can be reconstructed into 3D models, enabling precise visualization of anatomy and pathology [138]. Figure 7A shows a patient undergoing CT scanning (top) and the resulting abdominal cross‐sectional image (bottom) [133]. The integration of CT imaging is particularly valuable in in situ bioprinting for bone and calcified tissues, as its high effectiveness in visualizing rigid structures facilitates accurate construct evaluation [138]. In many situations, contrast agents are used in CT scans to enhance the visibility of soft tissues, which naturally have lower X‐ray absorption, by introducing substances that block X‐rays, making them more visible on the scan [139]. For example, Forget et al. used barium sulfate as a contrast agent, which was injected into the hollow channels of the bioprinted scaffold, and then imaged with CT to capture and visualize the 3D structure of the vessel‐like channels for design validation [140]. Gil et al. incorporated contrast agents into the bioinks used for 3D bioprinting, enabling enhanced X‐ray attenuation to improve the visibility and tracking of the scaffolds during CT imaging [141]. However, CT scans use ionizing radiation, which has the potential to cause biological effects in living tissue, and although the risk of developing cancer from exposure is generally small, it increases with the number of exposures over time [133, 142].

MRI is a medical imaging technique that uses strong magnetic fields and radiofrequency pulses to align and disturb protons in the body, generating detailed images based on tissue magnetic properties and chemical composition [143]. Unlike CT, MRI does not use ionizing radiation, making it safer for repeated imaging, especially in sensitive clinical applications. Figure 7B shows an MRI scanner (top) and a cross‐sectional MRI image of the body (bottom) [134]. MRI is a non‐invasive imaging technology that provides high‐contrast, high‐resolution images of soft tissues based on the magnetic properties of hydrogen atoms in the body, making it extremely useful for bioprinting by enabling the creation of accurate 3D models of anatomical structures [144]. For example, Mirdamadi et al. use patient‐derived MRI data sets to print a full‐size model of the human heart, as shown in Figure 7C, which illustrates the process from creating a 3D CAD model, refining it for printability, generating the G‐code print path, to ultimately producing the 3D printed model [86]. Similarly, Gonzalez‐Fernandez et al. utilized MRI to create 3D models of the scaphoid bone from patient scans, which are then converted into STL files and bioprinted to fabricate anatomically accurate, cell‐laden bone grafts for osteogenic applications [145]. In addition, MRI can be used as a quality control tool to assess the geometric fidelity of 3D printed scaffolds by evaluating parameters such as porosity, hole size distribution, and stackability across different bioprinted materials [146]. However, MRI has key limitations: high cost; long, noisy, enclosed exams that provoke claustrophobia and demand stillness; safety contraindications and artifacts from many implants and metal; intrinsically low bone signal; and warranting caution in early pregnancy [134, 143].

High‐resolution medical images are critical for accurate bioprinted models, yet CT and MRI typically offer only millimeter‐scale resolution, below the micrometer precision of most 3D bioprinters [147]. This gap has prompted interest in deep learning–based super‐resolution (SR), where neural networks generate high‐resolution outputs from low‐resolution inputs [148, 149]. SR has shown effectiveness in CT [150, 151, 152, 153] and MRI [154, 155, 156, 157], but its use in bioprinting remains limited due to constraints from printing modalities, bioink rheology, and crosslinking. Current research focuses on these bottlenecks, explaining the lack of direct SR applications in bioprinting. Still, with continued advances, SR integration is expected to grow increasingly impactful.

Optical Coherence Tomography (OCT) is a noninvasive imaging technique that uses low‐coherence light to capture cross‐sectional tissue images by detecting interference between backscattered and reference beams [158]. Focusing on microscale surface and subsurface details, OCT is well‐suited for layer‐by‐layer validation in bioprinting. Integrated into bioprinters, it provides real‐time, high‐resolution 3D imaging for quality assessment, dimensional validation, and defect detection. Figure 7D demonstrates an extrusion‐based bioprinting system integrated with swept‐source OCT that enables real‐time, non‐destructive, high‐resolution 3D monitoring and automated multi‐parameter evaluation of scaffold fabrication, significantly improving defect detection and process control [135]. Tashman et al. demonstrated that integrating swept‐source OCT with FRESH 3D bioprinting enables volumetric imaging and multi‐parameter assessment of bioprinted scaffolds, providing accurate detection of structural features and defects to enhance in situ process monitoring [159]. Notably, Yang et al. demonstrate that integrating 3D P‐OCT with a feedback mechanism enables real‐time detection of material deposition defects, adjustment of printing parameters, and defect repair to improve printing fidelity [160]. Furthermore, the Preceyes System integrates an OCT A‐scan sensor at the instrument tip specify the boundary of the operation site with high axial precision [161]. This serves as clear evidence that OCT holds strong potential for detecting defect sites in situ bioprinting. However, OCT is still limited to external surface imaging, restricting its use to skin‐related applications in Stages 1–2 (Figure 1), rather than deep in situ bioprinting in Stages 3–4

Structured‐Light Scanning (SLS) is an optical imaging technique that projects a patterned light grid or stripe onto a surface and analyzes its deformation via cameras to reconstruct 3D geometry [162]. The advantage of SLS lies in its ability to combine high‐speed and high‐resolution pattern projection with advanced imaging sensors, enabling the development of next‐generation 3D surface measurement systems that surpass existing technologies in speed, accuracy, resolution, modularity, and ease of use, while offering a more cost‐effective solution than many other imaging methods [163]. Compared to OCT, it offers lower resolution but enables faster, large‐area surface assessment at a lower cost, making it useful for urgent bioprinting tasks. SLS has been integrated into robotic and handheld in situ bioprinters: Zhao et al. used structured‐light scanning for real‐time wound mapping to guide robotic skin deposition (Figure 7E) [136], while Albanna et al. employed it for portable systems that capture wound geometry and enable precise skin cell deposition, accelerating healing in animal models (Figure 7F) [115]. However, like OCT, the inability of SLS to image internal structures restricts its utility in deeper or volumetric bioprinting applications. Currently, MIB is still an emerging field, with limited devices and research available. However, as the technology continues to evolve, we hope to see compact SLS devices integrated into MIS systems or flexible catheters, enabling direct scanning of internal organ wounds. This would not only open up the potential for more precise and effective applications in treating complex medical conditions but also allow for real‐time printing, enabling the immediate creation of biological tissues or therapeutic materials as wounds are scanned and analyzed.

Imaging choice for in situ bioprinting depends on defect size, depth, location, resolution needs, real‐time capability, and system compatibility. CT and MRI are preferred for deep or internal defects, offering volumetric subsurface data. OCT, as an optical method, is ideal for small, shallow, or layered defects, providing microscale resolution and real‐time monitoring to guide fine adjustments. SLS excels for large or external defects, such as skin wounds, capturing surface geometry quickly, which is critical in urgent cases. If developed to be compact in the future, SLS has huge potential for MIB, as it could be integrated into flexible soft robots, enabling real‐time scanning of internal organ wounds and allowing immediate printing decisions in emergencies. Integrating multiple modalities enables complementary strengths, ensuring optimal outcomes despite individual limitations.

### 4.2 Bioink Preparation Strategies

Bioinks are biomaterials formulated to encapsulate living cells, biomolecules, and extracellular matrix (ECM) components for use in bioprinting [5]. In the context of in situ tissue repair, bioinks serve as both the structural and biological foundation, enabling cells to survive, proliferate, self‐organize, and differentiate into functional, organ‐like structures. Based on origin and composition, biomaterials are classified into four categories: natural hydrogels, synthetic polymers, composites, and decellularized ECM (dECM). Natural hydrogel‐based bioinks are derived from biopolymers such as collagen [105, 164, 165], alginate [166], fibrin [167, 168], matrigel [169, 170], cellulose [171, 172], and chitosan [173, 174]. These materials are highly biocompatible and mimic the extracellular matrix, providing critical cues for cell adhesion, proliferation, and differentiation [175]. Synthetic bioinks are formulated from synthetic polymers such as polyethylene glycol (PEG) [176], polylactic acid (PLA) [177], and polycaprolactone (PCL) [178]. These materials offer customizable mechanical properties and degradation rates to support the structural integrity of the printed constructs [25]. Composite‐based bioinks combine natural biomaterials, which provide biocompatibility and cell‐friendly environments, with synthetic polymers that add mechanical strength and structural control. This balance ensures both biological functionality and printability for tissue constructs requiring soft cellular niches and stable architectures [25]. For example, silk fibroin/PEG composite bioinks enable self‐standing 3D structures with high printability and biocompatibility, supporting long‐term stem cell culture [179]. dECM‐based bioinks are developed by removing cellular components from native tissues while preserving the structural and biochemical features of the extracellular matrix. These bioinks offer a highly biomimetic environment, enabling cells to interact with tissue‐specific ECM cues and promoting tissue maturation that closely resembles in vivo conditions [180]. Some examples of dECM‐based bioinks include hydrogels derived from decellularized human kidney cortex [181], tissue‐specific dECM from adipose, cartilage, and heart [182], and fetal brain ECM for neural tissue engineering [183].

In situ bioprinting directly deposits bioinks into damaged tissues within dynamic, complex environments. This setting demands bioinks that function reliably under biological conditions while ensuring safety and therapeutic efficacy. Therefore, designing bioinks for this setting must address several key challenges.

First and foremost, bioinks designed for in situ bioprinting must satisfy a set of rigorous physiological and logistical criteria that distinguish them from conventional in vitro materials: (1) Physiological thermal stability: Unlike temperature‐controlled laboratory setups, the human body acts as a fixed thermal bath at approximately 37°C. Consequently, bioinks must maintain their rheological characteristics and not degrade or prematurely liquefy under physiological conditions. (2) Rapid in situ stabilization: In dynamic clinical environments where patient movement, respiration, or organ peristalsis is inevitable, rapid crosslinking is essential. The printed structure must adhere and stabilize immediately upon deposition to prevent displacement or deformation. (3) Biocompatibility and safety: Bioinks must be strictly non‐cytotoxic and non‐immunogenic to avoid provoking adverse immune responses or inflammation when applied directly to living host tissues. To avoid infection during clinical use, bioinks must undergo rigorous sterilization without adversely affecting the bioink [184]. (4) Clinical deployability: particularly for emergency trauma care or resource‐limited settings, bioink formulations should be portable, easy to prepare, and ready for immediate deployment without requiring complex infrastructure.

Beyond general physiological constraints, the physicochemical properties of bioinks must be strictly tailored to the specific bioprinting modality employed, as the governing physics of deposition vary significantly between techniques.

For extrusion‐based systems, which utilize pneumatic or mechanical pressure to deliver a continuous filament, the bioink must exhibit specific rheological properties, most notably shear‐thinning behavior. Specifically, shear‐thinning properties allow the bioink to reduce its viscosity under pressure for delivery through narrow channels or catheters, while its rapid post‐extrusion rheological recovery ensures immediate structural stability upon contact with the target tissue [185]. However, excessive extrusion pressure required to dispense viscous bioinks can induce high stresses that compromise cell viability [33].

In contrast, jetting‐based bioprinting operates by thermally or acoustically ejecting bioink droplets to construct a graft. This mechanism necessitates formulations in a liquid state with sufficiently low viscosity (typically 3–10 mPa·s) to ensure stable ejection from the nozzle orifice [91]. While this rheological requirement limits the range of printable materials compared to extrusion, the modality offers a distinct clinical advantage due to its non‐contact nature. Unlike contact‐based methods, jetting enables the precise deposition of hydrogel‐based bioinks, such as alginate‐based, collagen‐based, or gelatin‐based, onto non‐horizontal or irregular tissue surfaces [91]. This capability is of significant value for in situ applications, allowing for the repair of complex, curved anatomical defects where planar deposition is unfeasible. Consequently, this approach is predominantly utilized for fabricating thin, delicate tissue constructs, including [186], alveolar lung [108], and retina [187].

Alternatively, vat photopolymerization‐based bioprinting operates by utilizing light to solidify liquid bioinks into cell‐filled structures [93]. Since local variations in pH, fluid flow, or ion concentration can unpredictably affect chemical crosslinking in the in vivo environment, light‐induced strategies are often preferable for their speed and spatial precision [94]. Therefore, for this process to function effectively in situ, the bioink must be designed to solidify rapidly upon exposure. Nevertheless, this approach faces challenges regarding light penetration depth, as the light source must reach deep tissue layers to fully cure the construct [188]. Moreover, standard ultraviolet light can be phototoxic to exposed host tissues [94]. To mitigate these risks, advanced bioinks must be compatible with safer, visible light sources or utilize protective strategies.

### 4.3 Potential Soft Actuation in MIB

MIB inherits the catheter‐based architectures long used in MIS: a flexible shaft or working channel steers through tortuous anatomy while only the distal instrument (e.g., a micro‐printhead/nozzle, light guide, or curing module) acts at the target site. Because actuators must navigate with low interaction forces and minimal tissue trauma, power and bulk are typically proximalized or mounted away from the surgical field, and transmission occurs through soft or compliant structures. Soft robotics has therefore emerged as a strong candidate, mitigating the limitations of rigid robots. Five main actuation methods drive soft surgical devices: cable‐driven, magnetic, smart material, fluidic, and jamming material actuators.

#### 4.3.1 Cable‐Driven Actuation

**FIGURE 8:**
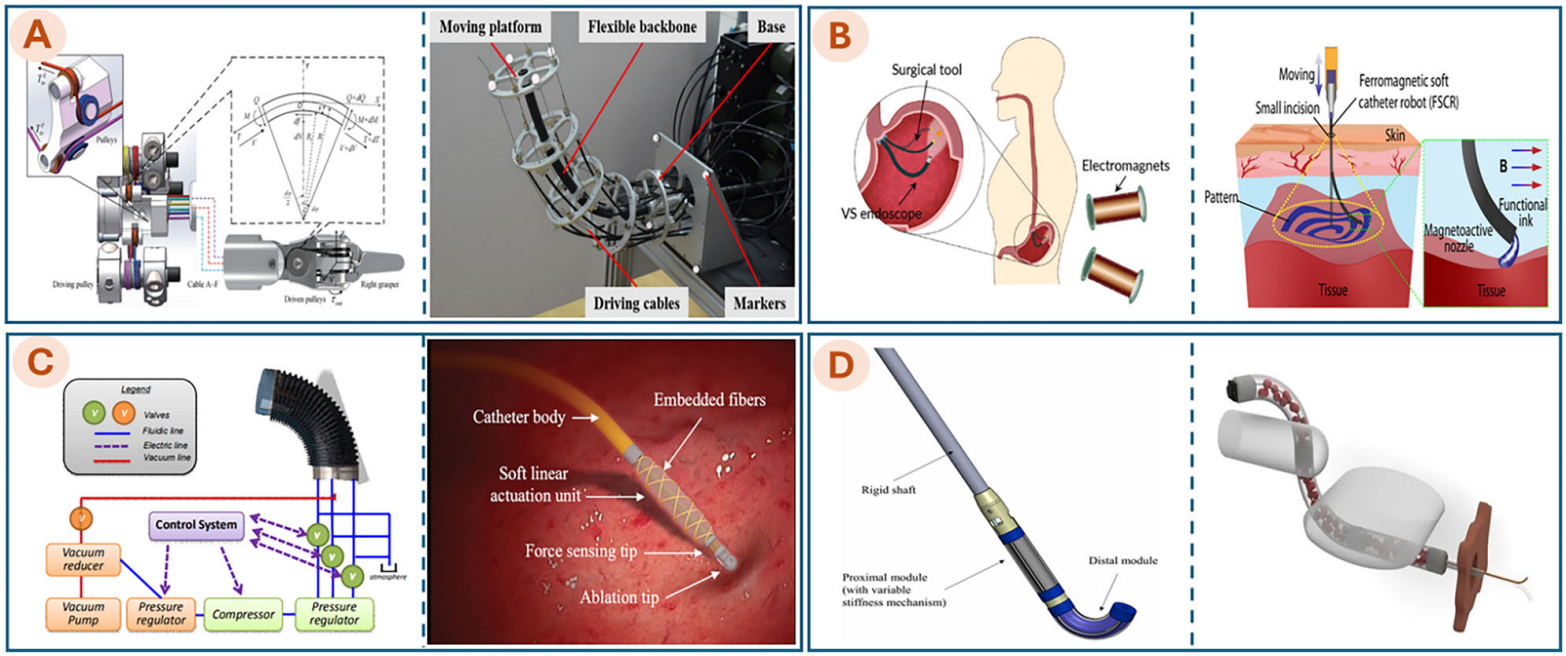
Potential soft actuations for minimally invasive procedures. (A) The cable‐driven actuation: ***Left***) Cable‐driven actuation transmits motion and force to the flexible tip via tensioned cables routed through pulleys. Reproduced with permission from ref. [189] Copyright 2017, Elsevier; ***Right***) Cable‐driven continuum robot with a flexible backbone. Reproduced with permission from ref. [190]. Copyright 2020, IEEE. (B) Magnetic‐driven soft actuation: ***Left***) Magnetic actuation controls the catheter's shape and movement by interacting with embedded magnetic particles in response to an external magnetic field. Reproduced with permission from ref. [191]. Copyright 2022, John Wiley and Sons; ***Right***) Ferromagnetic soft catheter robots for MIB. Adapted with permission from ref. [59]. Copyright 2021, Springer Nature. (C) Soft fluid‐driven actuation: ***Left*)** The control and actuation setup for the STIFF‐FLOP manipulator module. Reproduced with permission from ref. [69]. Copyright 2013, IEEE; ***Right***) A cardiac ablation catheter with an integrated soft fiber‐reinforced hydraulic actuator. Adapted with permission from ref. [192]. Copyright 2021, Mary Ann Liebert, Inc. (D) Jamming actuation: ***Left***) Representation of the two‐module STIFF‐FLOP manipulator attached to a rigid shaft used as support. The proximal module has been redesigned to lodge a variable stiffness system that can become rigid on demand and provide stability to the distal module. Reproduced with permission from ref. [193]. Copyright 2019, Frontiers; ***Right***) A granular jamming‐based flexible laparoscopic camera. Reproduced with permission from ref. [194]. Copyright 2013, King's College London.

The first category, cable‐driven actuation, has the benefits of miniaturization, high generated force, high safety, and low weight [195]. This method avoids the bulk and weight of onboard DC motors and is widely used to control the flexible tips of continuum and surgical devices, including the da Vinci and Medrobotics Flex systems [196]. Xue et al. used cable‐driven actuation to relay control inputs from motors through a network of pulleys and tensioned cables, steering and powering the flexible end‐effector tip of a laparoscopic surgical robot (Figure 8A‐Left) [189]. Furthermore, the flexible tip position of the catheter in radiofrequency ablation of cardiac arrhythmias is controlled by rotating/pushing/pulling wires located at the distal end of the catheter [197]. In cable‐driven actuation, the desired position or force is transmitted from outside driving mechanisms to distant tools via fixed points (pulleys) or flexible tubes (sheaths) [6]. While the tendon–sheath structure has been broadly used in robotic catheters and flexible endoscopic systems, most laparoscopic surgical systems utilize the cable‐pulley system as the main mode of transmission. The combination of cable‐driven mechanisms and other extrinsic actuation mechanisms, such as multi‐backboned structures [198] and concentric tubes [199], was also implemented in many surgical systems. However, cable‐driven actuation is generally expensive and suffers from mechanical limitations such as non‐linear friction, backlash, hysteresis, and poorly transmitted forces due to the need to pass tension wires through long and complex paths. To address this limitation, Shen et al. integrated a two‐variable POE kinematic model with the Gaussian Process Regression technique to compensate for tip errors and significantly improve the positioning accuracy of segmented cable‐driven continuum robots (Figure 8A‐Right) [190]. Although cables are pre‐tensioned to prevent slack, key mechanical limitations persist. Motion and output force depend on the distance between the end‐effector and the proximal power source, reducing responsiveness and accuracy [200]. Most cable‐driven systems, therefore, use open‐loop control with operator vision as feedback, making automation difficult.

#### 4.3.2 Magnetic‐Driven Actuation

Magnetic‐driven actuation has recently emerged for MIB systems [201]. The main structure of this actuation type is a composite between magnetic particles or purely permanent magnets and soft silicone elastomers. The actuator motion is driven by an external magnetic field that penetrates most materials through the use of magnetic fillers inside the actuation structure [202]. Therefore, it can wirelessly control the surgical tools without requiring a physical transmission link between the power source and the tools. For example, Zhao et al. used magnetic actuation to control the shape and movement of the catheter by applying an external magnetic field to interact with the embedded magnetic particle, enabling its use in MIS (Figure 8B‐Left) [191]. Zhou et al. utilized magnetic actuation to control the movement and printing process of a ferromagnetic soft catheter robot, which uses an external magnetic field to manipulate the catheter's position for in vivo bioprinting (Figure 8B‐Right) [59]. In surgery, magnetic generators placed near the patient apply force and torque to tools by modulating field strength and direction through electric current. Independent control of forces and torques enables complex motion without transmission along the device shaft, allowing smaller tool sizes than other actuation methods. Magnetic actuation has been widely applied in many preclinical surgical procedures, such as the tissue retractor in the human liver, a soft continuum robot [53], and an autonomous robotic intracardiac catheter [203]. Although these actuators offer faster responses compared to other types of actuators (up to 100 Hz) [204], they are not well‐suited for drug delivery, microsurgery, or microfluidics because the magnetic forces require a strong magnetic field generated from large magnetic coils (with electromagnetic actuation) or large permanent magnets. In addition, handling large permanent magnets or coils during surgery is challenging, especially near MRI machines or ferromagnetic materials, which introduce nonlinearities into the magnetic field [6].

#### 4.3.3 Smart Material Actuation

Smart material actuators are powered by electricity, which they convert into mechanical energy using materials such as fluids, polymers, paper, or gels [196]. Electric energy can be generated by compact controllers that regulate the magnitude, phase, and frequency of the electric current. This type of actuator has been used in many applications, including artificial muscles [205], small‐scale robots [206], and microfluidic systems [207]. Some of these smart materials depend on thermal energy to transform their state, such as shape memory alloys (SMA), which release stored elastic energy under temperature changes, as shown in live hinges on foldable systems and stiffness tuning layers for soft actuators [208]. Recently, Wang et al. devised a handheld “time‐share–driven” continuum robot in which SMA wires act as on‐demand clutches; a short voltage pulse contracts an SMA wire to lock a chosen bending module onto a common rotating shaft, allowing a single motor to steer multiple degrees of freedom [209]. Mattmann et al. developed a variable‐stiffness catheter whose shape‐memory‐polymer body can be actively heated by embedded resistive coils and actively cooled through internal micro‐water channels, enabling rapid, on‐demand rigidity tuning, a smart‐material actuation strategy [191]. Because of being biocompatible and dense, this actuation type has been applied to many surgical robotic systems for robotic catheters, endoscopes, and surgical graspers [210]. Other examples include smart materials that react to external stimuli such as temperature, light, or chemical compounds, as described in a recent publication [208]. However, these actuators often respond slowly and generate relatively low output forces compared with other actuation methods, which can limit their practicality for surgical robots; consequently, they are frequently used as auxiliary elements, for example, as clutches, latches, stiffness‐tuning layers, or release mechanisms, to trigger or modulate other actuation or control functions rather than serving as the primary drive source.

#### 4.3.4 Flexible Fluidic Actuators

Recently, flexible fluidic actuators or soft fluidic actuators have emerged as one of the most promising actuation methods for MIB devices [211]. Flexible fluidic actuators are compliant, inflatable structures activated by the application of fluid pressure. These include: hydraulic‐driven actuators [212, 213], which utilize pressurized liquids; pneumatic‐driven actuators [214], powered by compressed air; and vacuum‐driven actuators, which deform under negative pressure. The main components of fluidic actuators include a hollow deformable chamber, an inextensible constraint layer, and an active driver. Based on the structure of the deformable chamber and the constraint layer, when the active driver exerts pressure, fluidic actuators can generate a wide range of motions, such as elongation, rotation, or bending [208]. This actuator type also presented superior performance, including compliance, dexterity, high strain, high stress, and high energy efficiency [6]. Another significant benefit of the fluidic actuators is the simplicity in fabrication, which makes them affordable and disposable. With the exceptional safety during interactions with the human body, soft fluidic actuators pose great promise in the development of the next generation of medical tools [6], as well as being used in wearable haptic devices [215]. Figure 8C‐Left demonstrates the STIFF‐FLOP manipulator, operating on the principle of integrating soft pneumatic chambers for omnidirectional bending and elongation with a central granular jamming mechanism to enable tunable stiffness [69]. Kumar et al. developed a cardiac ablation catheter tip (Figure 8C‐Right) that combines a fiber‐reinforced hydraulic actuator for precise axial motion with an embedded liquid‐metal sensor for real‐time contact force feedback [192]. There are several noticeable existing devices, such as Decroly et al. [216] with a 2‐DOF bending actuator, Garbin [217] with a low‐cost disposable continuum endoscope, Caprata et al. [218] with a swallowable endoscopic capsule, and Fang et al. [219] with an MR‐safe soft robotic system. Soft fluidic actuators, while less frictional than cable‐driven ones, have several limitations, including larger size, lower force, and higher weight. Additionally, their viscoelastic and compliant properties lead to hysteresis and poor control accuracy, posing significant challenges [220]. Therefore, developing reliable hysteresis‐aware nonlinear models and corresponding closed‐loop control strategies is critical for the precise, repeatable operation of fluidic actuators in MIB applications.

#### 4.3.5 Jamming Material Actuators

A jamming actuator is a soft robotic mechanism that varies stiffness by transitioning flexible materials into a rigid state. Inside a membrane, grains, sheets, or fibers remain soft when unjammed but compact under vacuum pressure, ‘jamming’ into a stiff structure. This soft‐to‐stiff switching with minimal volume change makes them versatile for gripping, locomotion, and minimally invasive tools. The three main types of jamming actuators are granular jamming (utilizing particles such as sand or coffee), layer jamming (employing stacked sheets), and fibre jamming (utilizing bundled threads), each offering unique advantages depending on the intended use.

Granular jamming, the most common type, enables flexible designs with tunable stiffness for specific tasks. This adaptability makes soft robots highly efficient, especially in minimally invasive procedures and unstructured environments. For example, Brancadoro et al. [193] introduce a manipulator that uses fiber jamming for variable stiffness control, enhancing the STIFF‐FLOP manipulator's force application in MIS (Figure 8D‐Left); Jiang et al. [194] developed an octopus‐inspired robot arm that uses granular jamming to transition between flexible and rigid states for MIS (Figure 8D‐Right). However, the reliance on vacuum systems for the actuation introduces complexity and weight, and the behavior of the granular materials can sometimes be unpredictable, reducing stability and control [221].

### 4.4 Sensing Technologies

#### 4.4.1 Force Sensing

The absence of force sensing in minimally invasive and in situ bioprinting limits surgeons’ awareness of tool–tissue interactions, pushing them to rely on insufficient visual cues. This increases the risk of excessive force, causing tissue injury, perforation, or damage to fragile bioprinted constructs [228], and reduces accuracy in complicated tasks like narrow‐path navigation or layered fabrication. Moreover, it also hampers robotic/automated control, leading to an increase in cognitive workload on surgeons.

**FIGURE 9:**
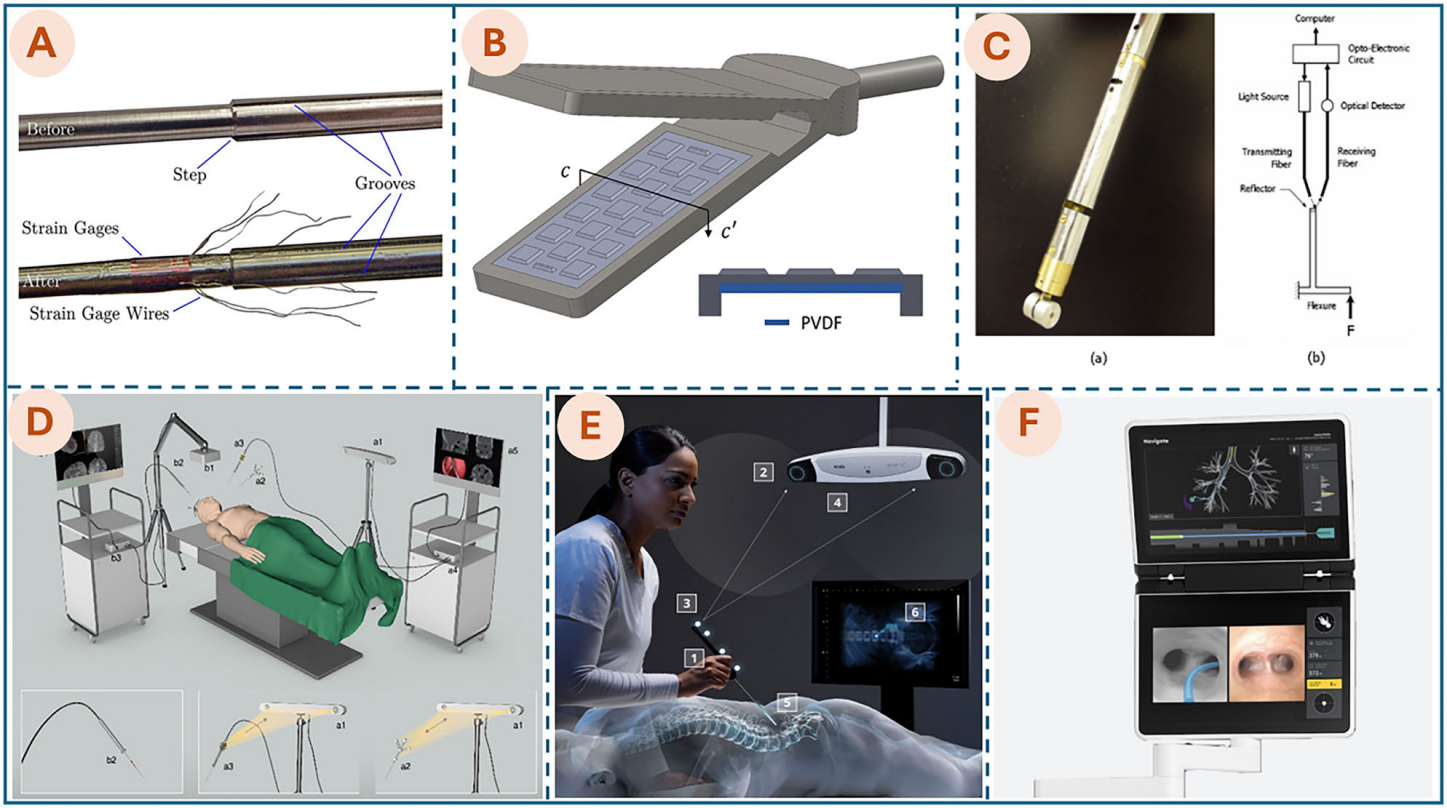
Sensing technology for MIS and MIB. (A) Examples of strain gauges in MIS instruments to measure forces. Reproduced with permission from ref. [222]. Copyright 2024, Wiley. (B) Mesa‐structured piezoelectric tactile sensor mounted on an endoscopic grasper for enhanced grip stability. Reproduced with permission from ref. [223]. Copyright 2024, Wiley. (C) An optical fiber sensor designed to perform tissue stiffness palpation. Reproduced with permission from ref. [224]. Copyright 2008, IEEE. (D) The components of optical (a1‐a5) and electromagnetic (b1‐b4) tracking systems are used for precise medical procedure guidance. Reproduced with permission from ref. [225]. Copyright 2019, IEEE. (E) The Polaris optical measurement system, where an optical tracker and markers on surgical instruments provide real‐time tracking and navigation during surgery. Image courtesy of Northern Digital Inc [226]. Copyright 2026. (F) The display of the Ion Intuitive platform uses real‐time shape sensing as a navigation system. Image courtesy of Intuitive Surgical, Inc [227]. Copyright 2026.

Strain gauges, made of thin metal foils, measure surgical tool forces by detecting resistance changes resulting from film deformation. Figure 9A shows the MIS instrument tube before (top) and after (bottom) applying strain gauges [222]. Even though each strain gauge is only able to measure one‐directional force, positioning multiple sensors at desired locations or using novel structures can achieve multi‐axis force sensing [229]. The strain gauge can be designed with a small scale and waterproof capability to integrate into surgical tooltips. Nevertheless, this type of sensor is normally interfered with by electromagnetic noise and temperature changes, preventing them from being used in clinical practice, where several imaging machines, such as MRI, are working [230].

In contrast with strain gauges, capacitive sensors can provide force measurement based on the capacitance change between two electrodes [231]. When force deforms the first electrode, it alters the electrode spacing or dielectric thickness. The applied force is then calculated from this dielectric variation using a mathematical relationship [232]. The sensitivity of this type of sensor depends on Young's modulus of the dielectric layer. Due to its simple fabrication and low cost, this type of sensor has been used in many surgical systems for disposable use. For example, capacitance sensors were attached to the surgical grasper to extract 3‐DOF force feedback [233]. A firm grasp for the twisting and handling of biological tissues was achieved by Qasaimeh et al. through the application of mesa structures on a flexible piezoelectric membrane (Figure 9B) [223]. Capacitive sensors offer greater stability and sensitivity than strain gauges in warm, wet environments but demand complex signal processing and specialized packaging, leading to higher cost and manufacturing complexity.

Optical sensors estimate contact forces up to 6‐DOFs by detecting light intensity or phase changes in flexible fibers attached to compliant structures. They perform well in magnetic environments with low hysteresis and good reproducibility. Figure 9C shows a bent‐tip fiber sensor using reflective light modulation to measure axial forces and tissue stiffness during MIS [224]. A 5.5 mm diameter miniature tactile sensor was designed to measure the tissue‐tool interaction force in a beating heart during mitral valve annuloplasty [234]. A force sensor that was fabricated by a 1 mm fiber Bragg grating (FBG) and a 3 mm long nitinol tube was used to detect the distal force in a tendon‐sheath mechanism [235, 236]. A fixed and sliding FBG sensor was proposed for the triaxial force sensing method in cable‐driven continuum robots [237]. However, its sensitivity and accuracy depend on the flexibility of the transmission fiber materials and the alignment with the compliant structure. Furthermore, the FBG is only able to measure large bending radii, preventing its use in miniature devices.

#### 4.4.2 Orientation Sensing and Tracking System

Pose sensing is essential for precision, safety, and effectiveness in MIS and MIB. In MIS, it enables accurate tool positioning in confined spaces, reducing errors and protecting delicate tissues. Real‐time feedback lets surgeons adjust movements precisely, improving safety and recovery. MIB demands even higher precision, as bioinks must be deposited at shifting or deforming sites. Pose sensing maintains tool alignment, ensuring accurate placement for tissue regeneration while enabling adaptive navigation and motion compensation. Optical and electromagnetic tracking (Figure 9D) are the main technologies used in current surgical navigation systems [225].

Optical Tracking Systems (OTSs) are widely used in biomedical applications for accurately tracking the position and orientation of surgical instruments or anatomical landmarks in real time. The fundamental principle behind OTSs is the detection of light, either emitted or reflected from markers that are fixed onto the tracked object. These systems typically operate in the visible or infrared spectrum, using either active markers (which emit light via LEDs) or passive markers (which reflect light from surrounding sources). An OTS is composed of three core components: (1) optical markers, either passive or active; (2) image sensors or cameras, usually equipped with filters to reduce ambient light interference; and (3) a central control unit that processes the image data to reconstruct the 3D pose of the object using triangulation or back‐projection algorithms [238, 239]. Well‐known commercial OTSs include Polaris Spectra and Polaris Vega by Northern Digital Inc. (NDI, Canada) [240], MicronTracker by ClaroNav (Canada) [241], and Vicon Vantage by Vicon (UK/USA) [242]. These systems are commonly used in clinical fields such as neurosurgery [243], orthopedics [242], and ENT surgery, where high spatial accuracy is critical. As shown in Figure 9E, the Polaris optical measurement system operates by using infrared light to track markers attached to surgical instruments, triangulating their 3D coordinates for real‐time navigation and visualization within a pre‐calibrated measurement volume. A key advantage of OTSs is their sub‐millimeter precision, which supports delicate and complex surgical procedures [244]. Additionally, they offer large working volumes and the ability to track multiple instruments simultaneously. However, a major limitation is their dependence on line‐of‐sight visibility. If the markers are occluded by tissue, tools, or the surgeon's hand, the system may fail to track accurately [225]. Flexible MIB tools, such as soft robotic needles or catheters, often operate deep in body cavities where tissue, blood, or instruments obstruct visibility. This can block optical signals, reducing accuracy or causing tracking failures. OTSs are therefore best suited for open or semi‐open environments, such as Stage 1 or 2 in situ bioprinting.

Electromagnetic Tracking Systems (EMTSs) are widely used in biomedical applications for real‐time tracking in minimally invasive and image‐guided procedures. They rely on field induction: a generator produces a low‐frequency magnetic field, and miniature sensors in surgical tools detect position and orientation by measuring induced voltage. An EMTS has three main parts: (1) a field generator establishing a reference system, (2) wire‐coil sensors integrated into or attached to instruments, and (3) a control unit that modulates the field and computes 3D pose. Unlike optical systems, EMTSs do not require a line‐of‐sight, which makes them particularly suitable for tracking flexible or inserted instruments within the human body, such as catheters, needles, or endoscopes, during procedures like endoscopic surgery, catheter navigation, or spinal interventions [225]. Notable commercial EMTSs include Aurora by Northern Digital Inc. (NDI, Canada) [245], trakSTAR by Ascension Technology (USA), and Liberty by Polhemus (USA) [225]. They provide real‐time, low‐latency feedback and can track multiple sensors across large volumes. However, they are prone to metal interference and magnetic noise, reducing accuracy, and offer fewer degrees of freedom than optical systems, limiting precision in complex tasks [246]. EMTSs are particularly advantageous for minimally invasive procedures, including minimally invasive in situ bioprinting, where tools are maneuvered through small incisions or narrow channels. Unlike optical systems, EMTSs are not hindered by obstacles such as blood, tissue, or surgical instruments, allowing them to function effectively in confined, occluded environments. In minimally invasive bioprinting, EMTSs enable precise tracking of soft robotic tools, such as catheters or flexible robotic needles, even when they operate deep inside body cavities.

Fiber Optic Shape Sensing (FOSS) is an emerging technology increasingly used in MIS and bioprinting, where precise monitoring of flexible instruments is critical. Unlike OTSs and EMTSs, which track only specific points, FOSS provides full‐shape information of surgical tools. A system typically includes: (1) optical fibers with strain sensors (e.g., FBG) that detect strain and curvature, (2) an interrogation unit that converts reflected light into strain data, and (3) a processing unit that reconstructs the 3D shape using algorithms [247]. The principle of FOSS is based on monitoring changes in the wavelength of light reflected by the FBGs when the fiber undergoes deformation, allowing for continuous, real‐time tracking of the instrument's shape and position in dynamic environments [248]. FOSS has been applied in many surgical instruments, such as colonoscopes for real‐time shape tracking [249], flexible needles for 3D steering [250, 251], flexible instruments for minimally invasive surgical systems [252], and active catheters for intravascular interventions [253]. As shown in Figure 9F, the Ion Intuitive system displays a complete view of the catheter, with real‐time shape sensing providing visual positioning and orientation, helping guide navigation during robotic‐assisted bronchoscopy procedures [227]. FOSS provides high precision, real‐time shape tracking, and immunity to electromagnetic interference, making it especially useful in strong magnetic fields like MRI‐guided procedures. It is compact, flexible, and biocompatible, enabling integration into small, deformable surgical tools [254], which is crucial for precise navigation and real‐time monitoring during MIS and MIB. Several emerging companies, including FBGS [255], TSSC [256], and Intuitive [227], are actively developing commercial FOSS solutions, accelerating the translation of this technology into clinical applications. Integrating fiber optic sensors with imaging gives FOSS a clearer, more accurate view of instrument position, reducing procedure time, complications, and recovery periods for better outcomes. High costs of sensors and interrogation systems may limit adoption in resource‐limited settings, yet FOSS remains a promising, actively researched technology for precision in medical robotics.

### 4.5 Modeling Techniques

#### 4.5.1 Kinematic Modeling and Mapping of Flexible Robots

Flexible robots, commonly referred to as continuum manipulators, are increasingly central to minimally invasive procedures due to their ability to navigate complex anatomical pathways safely. Unlike rigid‐link robots, continuum robots rely on distributed flexibility, which requires precise kinematic modeling to control the end‐effector's position and orientation while accounting for both internal actuation and external forces [257, 258]. Kinematic mapping is particularly critical for tasks such as endoscopic surgery and in situ bioprinting, where the external forces at the tool tip are typically low.

Continuum robot kinematics are often described using either constant‐curvature (CC) or variable‐curvature (VC) models. CC models simplify the robot backbone as a series of arc segments with fixed parameters, while VC models employ mathematical functions to describe elastic deformations along the backbone [259]. Most existing designs adopt the piecewise constant‐curvature (PCC) approximation due to its analytical simplicity and compatibility with various mechanical topologies [260, 261]. PCC kinematics assume (1) arc‐shaped bending and (2) negligible gravity [262]. These models involve three kinematic spaces: actuator space, configuration space, and task space. Two mappings are essential: (a) robot‐specific mapping from actuator to configuration space, and (b) robot‐independent mapping from configuration to task space [198]. Arc parameters (curvature, plane angle, arc length) define configuration space and vary by actuator type, such as cable length, pneumatic pressure, or tendon displacement. A detailed review of methods to accomplish forward kinematics can be found in [260].

Inverse kinematics maps a desired task‐space pose to configuration‐space arc parameters. Approaches include closed‐form geometric solutions, often using rigid‐link analogs, subsequently converted to arc parameters [261]. Jacobian‐based methods allow consideration of actuator constraints, adjusting the robot from any initial configuration to a target posture [263]. The actuator‐specific mapping from configuration to actuator space varies depending on whether the system uses push rods [264], cables, or pneumatic systems [265].

While constant‐curvature kinematic models are widely used due to their simplicity, they often overlook physical phenomena such as gravity, internal friction, and tissue interaction factors that can significantly impact robotic performance in delicate tasks like 3D in situ bioprinting. These limitations can be mitigated using redundant actuation and real‐time shape sensing to enable closed‐loop control [266]. Techniques include computer vision‐based shape tracking and visual servoing [267], as well as embedded strain gauges and fiber‐optic sensors, which provide real‐time feedback without line‐of‐sight constraints. Intraoperative imaging modalities (e.g., CT, MRI) are also invaluable for monitoring robot configuration and tissue interaction [268]. Furthermore, a growing area of research involves developing dynamic models that account for time‐dependent deformation and interaction forces. Such models are critical for enabling predictive and precise control of continuum robots navigating complex anatomies during minimally invasive in situ tissue fabrication [269].

#### 4.5.2 Nonlinear Hysteresis Model for Soft Actuators

Soft actuators are a core component of continuum robots used in MIB due to their compliance, safety, and ability to conform to anatomical constraints. While modeling efforts in soft robotics have often focused on cable‐driven or ferromagnetic actuation, fluid‐driven artificial muscles (FDAMs) have gained attention for their miniaturization potential, high compliance, and compatibility with imaging modalities such as MRI. Despite these advantages, FDAMs face challenges related to open‐loop control inaccuracies and hysteresis [220].

Hysteresis is a phenomenon where the output of a system depends not only on the current input but also on its previous history. In the case of FDAMs, this is caused by several factors, including memory effects, friction between the structural layers of the muscle, and elastic deformation of the soft materials used in the muscle. The memory effects result in the output signal highly relying on not only the current input but also the previous history of the output, making it difficult to accurately control the motion of FDAMs [270]. Accurately modeling hysteresis is therefore essential, especially in minimally invasive surgical settings where sensor integration is limited.

Several phenomenological models have been explored for hysteresis modeling, including Prandtl‐Ishlinskii, Preisach, Maxwell‐Slip, Krasnoselskii‐Pokrovskii, Duhem, and Bouc‐Wen models [271]. Among these, the Bouc‐Wen model is an effective method for various systems due to its lower complexity in both implementation and computation. This method can capture a variety of hysteresis profiles correctly, and it is also well‐known in civil and mechanical engineering [272]. In addition, it is controllable for the amplitudes and shapes of the hysteresis loops with smooth transition phases from positive velocity to negative velocity and vice versa. It also allows inverse modeling, enabling the design of hysteresis compensators for tendon‐driven or soft actuated mechanisms. However, this approach involves a trade‐off between the number of variables used and the achievable accuracy [273].

While closed‐loop control improves precision, it is often limited in surgical systems by sensor integration constraints, sterilization requirements, and size limitations. Real‐time feedback may introduce latency, complicating haptic control. Consequently, open‐loop, hysteresis‐based feedforward control remains practical and effective for many surgical soft robotic systems, including MIB platforms. The integration of kinematic modeling, dynamic prediction, and hysteresis compensation is crucial for soft continuum robots in MIB. Together, these approaches enable predictive, precise, and safe manipulation, allowing soft robotic systems to deposit cells and biomaterials in situ with high fidelity while adapting to the complex mechanical environment of internal organs.

### 4.6 Controlling Techniques

Control techniques in minimally invasive bioprinting underpin both navigation and deposition. During navigation, controllers translate surgeon or algorithmic commands into stable, accurate motion while compensating for uncertainties such as tissue motion, tool deflection, nonlinear dynamics, delays, and variable loading. Once the target site is reached, control becomes increasingly bioprinting‐centric, focusing on toolpath execution, surface registration, and coordinated regulation of printing parameters (e.g., nozzle speed, nozzle‐surface distance, and extrusion pressure) to ensure precise placement and gentle tool–tissue interaction. Accordingly, this section reviews control strategies spanning teleoperation and feedback‐assisted human‐in‐the‐loop schemes, as well as closed‐loop, sensor‐fusion, and emerging learning‐based approaches that improve robustness and precision for in situ printing tasks.

Most clinically deployed platforms remain strongly human‐in‐the‐loop, meaning the surgeon continuously receives intraoperative feedback, primarily visual, and in some systems, additional sensory cues, and uses it to interpret the surgical situation and update commands in real time. Real‐time visualization via endoscopic or camera‐based imaging, therefore, plays a central role in helping surgeons orient instruments, judge proximity to anatomy, and continuously adjust tool pose. At the most basic level, the surgeon's master inputs are transferred almost directly to the slave robot such that the instruments mirror the operator's hand motions. This mode is intuitive and clinically well established, but feedback is typically dominated by endoscopic vision; without reliable force/contact feedback, performance can be sensitive to tissue motion, instrument deflection, and poorly regulated interaction forces. These issues become especially critical for in situ bioprinting, where high‐precision placement and gentle, well‐controlled tool–tissue interaction are essential to prevent tissue trauma and maintain print fidelity.

Beyond visual feedback, more advanced systems incorporate additional sensing and feedback modalities to enhance intraoperative awareness and precision, including force/tactile cues, orientation sensing, and shape estimation. Such feedback can improve situational awareness, help regulate interaction forces, and reduce the risk of tissue trauma, capabilities that are particularly valuable for delicate manipulation and deposition tasks. Clinically oriented examples include systems integrating tactile or haptic feedback and force‐related cues, such as neuroArm [278], Senhance [279], and Da Vinci 5 Surgical System [280]. In ophthalmic microsurgery, the Preceyes System provides auditory warnings when the instrument approaches the retina and includes an emergency retraction mechanism for rapid tool withdrawal [161]. Several platforms also implement tremor‐filtering and motion‐stabilization functions to mitigate surgeon hand tremor and improve fine control, as reported for Flex, Preceyes, Zeus, and Da Vinci Surgical System [281].

**FIGURE 10:**
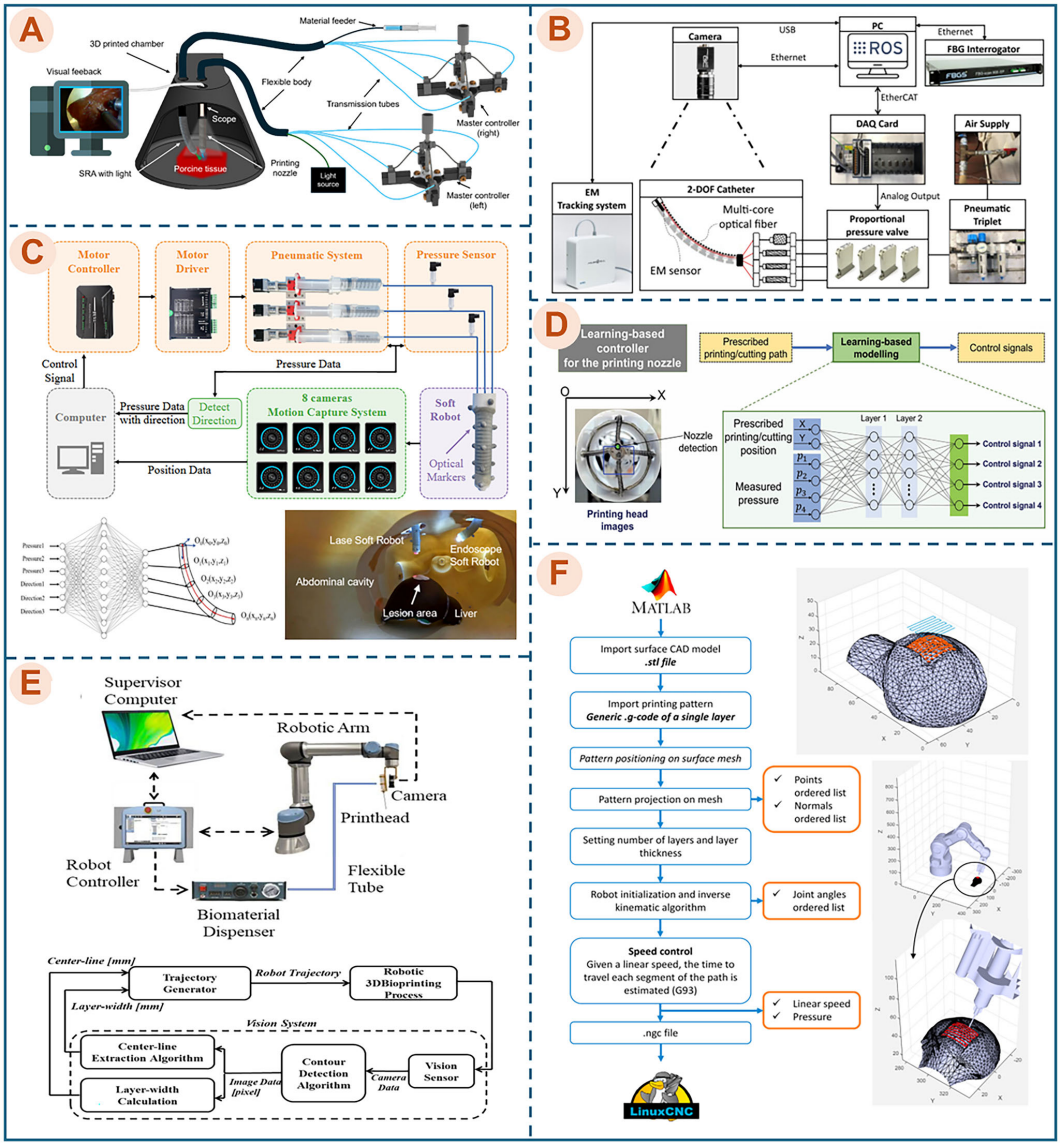
Examples of emerging controlling strategies in surgery and bioprinting. (A) A motor‐free soft robotic system for minimally invasive procedures. Reproduced with permission from ref. [23]. Copyright 2025, John Wiley and Sons; (B) Control and actuation system for the two‐DOF catheter system. Reproduced with permission from ref. [274]. Copyright 2023, IEEE; (C) Schematic outline of the hysteresis‐aware soft‐robot platform. Reproduced with permission from ref. [275]. Copyright 2025, arXiv; (D) A schematic outline of an ML‐based control system for a flexible robotic in situ bioprinting. Adapted with permission from ref. [10]. Copyright 2023, John Wiley and Sons; (E) A schematic setup of a vision‐based robotic bioprinting and the overall framework of the path correction process. Reproduced with permission from ref. [276]. Copyright 2024, Springer Nature; (F) A path planning algorithm developed in MATLAB for an in situ bioprinting robot. Reproduced with permission from ref. [277]. Copyright 2021, IEEE.

Recently, an emerging direction explores control concepts for soft robotic manipulators using hydraulic master–slave architectures. In these systems, user input is transmitted through fluidic coupling to achieve multi‐DoF teleoperation without electronics or electrical actuation at the slave side. Nguyen et al. demonstrated a motor‐free master–slave strategy where a handheld joystick (master) hydraulically drives a soft robotic manipulator (slave) (Figure 10A), enabling intuitive motorless actuation for surgical and bioprinting tasks [23]. Similarly, Zhu et al. employed a delta‐structured master device that hydraulically transmits user input to control dual soft arms, enabling intuitive and accurate teleoperation [52]. These architectures offer compelling advantages in weight, compactness, cost, and MRI compatibility, and they highlight a credible pathway toward lower‐cost platforms that could broaden access to minimally invasive care. Nevertheless, limited sensing and incomplete closed‐loop correction can restrict precision and safety, motivating systematic evaluation of tracking accuracy, force regulation, failure modes, and clinically appropriate sensing strategies for in vivo deployment.

Soft continuum robots are highly promising for MIB (as discussed in Section 3). Yet these same properties create major control challenges: pronounced nonlinearities, hysteresis, and time‐dependent dynamics complicate modeling and degrade tracking performance if not compensated. As a result, soft‐robot control increasingly relies on a combination of sensing, robust control design, and data‐driven compensation to maintain accuracy under uncertainty and changing contact conditions. To capture complex behaviors and enhance adaptability, recent hybrid strategies combine model‐based methods with machine learning. For example, a system (Figure 10B) uses FBG sensors and electromagnetic (EM) tracking, with an artificial neural network (ANN) estimating catheter shape in real time. Combining these complementary signals improves state observability and supports more robust closed‐loop control under deformation and occlusion [274]. Within this category, reinforcement learning (RL) has emerged as a powerful AI approach, enabling robots to learn control policies through trial‐and‐error with reward feedback [282]. Figure 10C shows a system where a pneumatic manifold, pressure sensors, and motion‐capture data feed a hysteresis‐aware neural network and RL algorithm for accurate trajectory tracking [275]. Shin et al. further developed RL and learning‐from‐demonstration algorithms for autonomous soft tissue manipulation using vision feedback, where the latter reduced training time and improved accuracy, achieving precise and reliable tissue positioning to advance surgical automation [282]. Beyond control techniques for precise navigation, Figure 10D shows a system that used a two‐hidden‐layer neural network trained with Bayesian regularization backpropagation, mapping the prescribed nozzle position (X, Y) and measured pressures (p_1_–p_4_) of hydraulic soft artificial muscles to the corresponding displacement of syringe plungers (l_1_–l_4_), improving nozzle trajectory tracking for precise printing [10].

Once the instrument is navigated to the target site, control for in situ bioprinting becomes increasingly toolpath‐centric, emphasizing printhead path planning and execution, including registration to the tissue surface, trajectory generation (e.g., from CAD), and coordinated control of printing parameters, with real‐time correction when available. For instance, Barjuei et al. proposed a vision‐based toolpath compensation approach (Figure 10E) in which a camera monitors the printed filaments, quantifies the deviation between the actual deposition path and the planned trajectory, and then updates the robot reference path (e.g., via centerline/width extraction) for subsequent printing, thereby improving trajectory tracking accuracy and reducing geometric errors in robotic bioprinting [276]. Notably, Fortunato et al. developed IMAGObot, a 5‐DoF platform for in situ bioprinting on irregular surfaces (Figure 10F), where CAD patterns are processed in MATLAB to generate surface‐adapted G‐code that coordinates robot motion, including tool orientation to follow the surface, with printing settings such as speed and extrusion pressure, supporting more consistent deposition on curved substrates [277].

Overall, control in minimally invasive bioprinting is evolving from vision‐dominant teleoperation toward feedback‐rich and learning‐enabled strategies that tightly couple state estimation, motion control, and toolpath execution. For soft continuum systems, the central challenge remains achieving repeatable, safety‐aware tracking under nonlinear and hysteretic dynamics and uncertain tissue contact. Future progress will likely depend on integrating multimodal sensing, robust control, and real‐time toolpath correction to ensure high‐precision placement and gentle interaction under realistic intraoperative disturbances.

## 5 Future, Potentials of Minimally Invasive Bioprinting

### 5.1 Navigation for Precision Delivery

Navigation is a core requirement in robotic‐assisted minimally invasive bioprinting because the device must traverse long, tortuous, and fragile anatomical pathways to reach deep defect sites with minimal trauma. Unlike open procedures, MIB operates with limited line‐of‐sight and under continuous tissue deformation and physiological motion, so navigation must be both safe and precise to achieve high‐precision deposition at the target. Building on the enabling technologies discussed in Section 4, this section outlines a practical navigation stack that combines imaging‐based planning, soft continuum actuation, tracking, feedback sensing, and advanced control.

Preoperative and intraoperative imaging technologies (Section 4.1) provide the initial anatomical context for navigation, enabling coarse planning of the target location and the access route. In practice, imaging helps define where the defect site is, which anatomical corridor the robot should follow, and which regions should be avoided, thereby providing a global map before active steering begins in confined anatomy.

In minimally invasive bioprinting, soft actuation for soft continuum robots (Section 4.3) provides the physical basis for navigation because these devices combine high flexibility with a high‐DoF motion, enabling dexterous steering through tortuous anatomy. This flexibility allows steering through curved lumens and reaching deep targets while maintaining compliant interaction with surrounding tissues.

Beyond mechanical capability, reliable navigation requires knowing where the device is inside the patient. Tracking systems (Section 4.4) play a central role by providing global spatial feedback for guidance, such as instrument position and orientation relative to anatomical targets, enabling accurate steering when direct visualization is limited in deep, confined environments. In addition, safe delivery also depends on how the tool interacts with tissue and what configuration the flexible tool has assumed along its body. Therefore, feedback sensing (Section 4.4), for example, force sensing to manage contact loads, and shape/configuration feedback such as fiber‐optic shape sensing (FOSS) for reconstructing tool shape in real time, supports both human‐in‐the‐loop operation and assisted/autonomous control by enabling timely correction and constraint enforcement.

Finally, modeling and control (Sections 4.5 and 4.6) integrate actuation, tracking, and sensing into a closed‐loop navigation capability. Kinematic/dynamic models relate actuator inputs to motion, while advanced control strategies fuse tracking outputs and sensor feedback to compensate for nonlinearities, hysteresis, and uncertainty in vivo, improving stability, reducing drift and overshoot, and enhancing repeatability for precision bioink placement.

Taken together, precision navigation for MIB should be viewed as an integrated pipeline rather than a single component: imaging establishes the initial map and target context, actuation provides physical access, tracking provides global localization, sensing provides safety‐ and configuration‐critical feedback, and modeling/control integrates these signals to execute reliable steering in vivo. Looking forward, tighter fusion of multimodal imaging, tracking, and shape/force feedback with intelligent control can reduce operator workload while improving repeatability, particularly in deep, occluded environments. Ultimately, advances in this navigation stack will be decisive for translating MIB from proof‐of‐concept demonstrations into clinically robust, safe, and accurate therapies.

### 5.2 4D Bioprinting for Adaptive Implants

4D bioprinting is an emerging biofabrication paradigm that expands traditional 3D bioprinting by adding time as a functional dimension. While 3D bioprinting allows the creation of static, cell‐laden structures by layering biomaterials and living cells, 4D bioprinting enables these printed structures to change their shape, function, or behavior over time in response to external stimuli such as temperature, moisture, pH, light, or magnetic fields [283, 284]. This adaptability is achieved using stimuli‐responsive (smart) biomaterials with intrinsic capabilities to self‐assemble, fold, expand, or contract after fabrication.

One promising application of 4D bioprinting is the development of smart therapeutic carriers that remain dormant until triggered by physiological cues. For example, a printed patch may remain closed under normal conditions but open to release antibiotics when infection‐induced acidosis lowers tissue pH [271]. Similarly, Goyanes et al. employed single‐filament FDM to 3D print paracetamol‐loaded enteric tablets whose delayed‐release profiles can be tuned for patient‐specific oral therapy [285]. Okwuosa et al. advanced this concept with dual‐nozzle FDM systems to produce shell‐core enteric tablets tailored for individualized drug delivery [286].

Beyond drug delivery, 4D bioprinting holds great promise for adaptive implants such as stents and scaffolds. Miniaturized stents fabricated from shape‐memory biomaterials could be delivered through catheters and later activated by physiological or externally applied stimuli to self‐expand into their functional geometry. Bodaghi et al. introduced a 4D‐printed shape‐memory lattice with reversible expansion and contraction, proposing its tubular variant for minimally invasive stent applications [287]. Next, Wei et al. developed a direct‐write 4D‐printing strategy to fabricate magnetically responsive shape‐memory scaffolds that remotely self‐expand as intravascular stents [288].

For internal organ repair, 4D bioprinted implants could be deployed via flexible catheters or soft continuum robots and then self‐assemble, conform, or expand directly at the defect site. Such adaptive behavior could restore mechanical integrity, promote tissue integration, and adapt to dynamic physiological environments without invasive fixation. Looking ahead, the convergence of adaptive biomaterials, patient‐specific cell sources, and precision delivery platforms promises truly personalized, living implants that dynamically respond to each individual's healing process, potentially lowering rejection rates and enhancing long‐term organ function.

### 5.3 Integration with Organ‐on‐Chip Platforms

Organ‐on‐a‐chip (OoC) systems are microfluidic devices designed to replicate the key structures and functions of human organs within a controlled environment. These devices typically integrate bioengineered tissues, natural tissues, or organ parts into a controlled microenvironment that allows real‐time monitoring and manipulation of biological processes. They are designed to mimic organ‐level physiological responses and are used as in vitro models for studying human biology, drug testing, and disease modeling [289]. While conventional fabrication techniques can reproduce some microarchitectures, their limited resolution and reproducibility restrict the complexity and biomimicry of the models. Bioprinting has recently emerged as a powerful tool for OoC fabrication due to its ability to precisely deposit multiple cell types and biomaterials with high spatial resolution and reproducibility.

Bioprinting streamlines OoC construction by providing: (1) sterile, automated cell delivery that minimizes contamination and human error; (2) near‐instant cell immobilization, eliminating the 6–8 h adhesion delay typical of manual seeding; (3) high experimental reproducibility and throughput because of computercontrolled dispensing; (4) micrometrescale spatial patterning that multiple cell types can be positioned with micrometre accuracy to recreate organspecific architectures (e.g. branched vasculature) which pipettes or pumps cannot achieve; (5) precise tuning of celltocell ratios, whereby each printed droplet can be formulated to contain an exact number of cells ranging from single‐cell deposits to small multicellular clusters so that researchers can mix, for example, two endothelial cells for every one pericyte or any other ratio needed to mimic natural tissue; (6) 3D bioink printing with advanced biomimicry, combining layerbylayer construction of perfusable microvasculature, barrier interfaces (e.g. blood–brain, gut–vascular), and complex tissue architectures in a single workflow. These capabilities have already enabled the fabrication of OoC models for diverse tissues, including the liver [170, 290, 291], placenta [292], blood vessel [293, 294], and myocardium [295], demonstrating its versatility in recapitulating organ‐specific architecture and function.

Looking forward, the convergence of bioprinting and OoC technologies promises to generate dynamic, patient‐specific microphysiological systems with unprecedented predictive accuracy. In the context of MIB, OoC platforms may serve as preclinical testing beds to optimize bioinks, adaptive implants, and delivery strategies before clinical application. Ultimately, these integrated systems could accelerate the translation of MIB from experimental settings to personalized therapies, bridging the gap between benchtop models and in vivo applications.

### 5.4 Artificial Intelligence in Minimally Invasive Bioprinting

Bioprinting typically involves three main stages: pre‐printing, printing, and post‐printing, as outlined in Section 3.1. Artificial intelligence (AI) has shown transformative potential across all these stages, offering capabilities such as design optimization, process control, defect detection, and outcome evaluation. A wide spectrum of models has been employed, spanning traditional machine learning approaches such as decision trees (DT), random forests (RF), k‐nearest neighbors (kNN), and regression algorithms, as well as advanced deep learning techniques including multilayer perceptrons (MLP) and long short‐term memory (LSTM) networks.

In the pre‐printing stage, AI has been employed to optimize bioink formulations and predict their biological and mechanical performance. For example, Lee et al. demonstrated a machine learning–based strategy to design 3D‐printable bioinks, revealing a universal relationship between elastic modulus, yield stress, and printability, thereby providing predictive guidelines for developing biocompatible hydrogel formulations [296]. Similarly, Chen et al. used DT, RF, and DL models to delineate a “printability window”, guiding formulation selection [297], while Sarah et al. applied polynomial fit, DT, and RF models to estimate bioink viscosity based on component weights and shear rate, accelerating formulation optimization [298]. Xu et al. developed an ensemble ML model combining NN, ridge regression, kNN, and RF to forecast cell viability in stereolithography‐based bioprinting [299], and Shin et al. applied supervised ML to predict droplet volume from rheological and process parameters, enabling real‐time parameter adjustment [300]. Beyond materials, Oncu et al. integrated ANN and CNN models to assess scaffold biocompatibility from slicer‐generated design images, reducing trial‐and‐error and material waste [301]. In addition, AI‐driven super‐resolution applied to imaging modalities such as CT and MRI (see Section 4.1) can enhance pre‐printing workflows by generating higher‐fidelity anatomical datasets for constructing accurate patient‐specific models.

During the printing stage, AI contributes both to the control of soft robotic manipulators and to real‐time monitoring of printed structures. As noted in Section 4.6 (Figure 10B–D), AI‐driven strategies are essential for managing the nonlinear mechanics of soft robotics, which hold great promise for MIS and MIB, enabling accurate and reliable motion control. Beyond robotics, AI algorithms have been widely applied for in‐process monitoring, where they detect defects, predict failures, and adjust printing parameters to improve accuracy and consistency. For example, a CNN‐based quality control loop has been implemented in extrusion bioprinting to provide real‐time monitoring, error classification, and automatic parameter optimization [302]. Deep CNNs trained on print images have achieved high accuracy in detecting anomalies such as discontinuity, irregularity, and nonuniformity [29]. In material jetting, Segura et al. developed a tensor time‐series deep learning framework to forecast droplet morphology evolution, allowing early detection of jetting instabilities [303]. Recently, Huang et al. applied supervised ML models to predict the number of cells in inkjet‐printed droplets based on velocity profiles, enabling parameter tuning to ensure consistent deposition [304].

In the post‐bioprinting phase, AI contributes to the maturation of printed constructs, which require tightly controlled environments for nutrient delivery, biomechanical stimulation, and cell–cell interactions. AI models can facilitate this stage by analyzing multimodal data to monitor cell growth, detect structural defects, and predict maturation trajectories, while also optimizing culture conditions such as medium composition and stimulation regimes. Optimization of co‐culture conditions, essential for organ‐level constructs, is another area where AI may reduce the reliance on time‐consuming trial‐and‐error [186, 305, 306]. Current limitations include scarce standardized datasets and restricted availability of a real‐time feedback system. However, emerging organ‐on‐a‐chip, microfluidic platforms and the integration with bioprinting (as demonstrated in Section 5.3) promise richer data streams that could enable AI‐driven optimization of co‐culture conditions and accelerate functional maturation.

#### 5.4.1 AI Explainability and Biosafety Assurance

While AI‐driven approaches offer superior capabilities in compensating for physiological motion and optimizing printing paths, their application in clinical MIB faces a significant hurdle: the “black‐box” nature of the algorithms. Unlike traditional deterministic control systems, deep learning models often function as stochastic algorithms that rely on probabilities, lacking transparency in how specific surgical decisions are reached. This inexplicability hampers the surgeon's ability to interpret the model's intent, creating a barrier to clinical trust and certification.

To address these biosafety concerns, a multi‐layered safety strategy is required. First, the integration of Explainable AI (XAI) is emerging as a critical requirement to make the robot's decision‐making process transparent and understandable to human operators [307]. Second, to mitigate the inherent uncertainty of probabilistic AI, the system must incorporate deterministic safety constraints. In this hierarchy, the AI optimizes performance within a defined safe zone, while the deterministic layer acts as a “fail‐safe” mechanism to prevent the system from exceeding physical safety boundaries.

Operationally, these technical safeguards must function within a strict ‘Human‐in‐the‐loop’ architecture. In this framework, AI serves in a supportive role rather than as a fully autonomous agent, tasked with precision‐enhancing sub‐tasks, such as real‐time tremor compensation, nozzle movement correction, automatic distance maintenance, and print error detection. The ultimate decision‐making authority and supervision remain vested in the surgeon, who retains the power to validate each step and intervene immediately.

In recent years, specialized organizations have been established to address the governance of advanced AI models, with the US AI Safety Institute serving as a prominent example [308]. These emerging regulatory bodies emphasize the necessity of implementing validated assessment tools and conducting rigorous pre‐release evaluations to identify and mitigate high‐consequence risks. Aligning with these evolving standards will be essential for future MIB systems to navigate the regulatory landscape and ensure safe clinical deployment.

Altogether, AI is poised to be a cornerstone technology for minimally invasive bioprinting, enabling adaptive control of robotic delivery systems, predictive optimization of bioink formulations, and intelligent post‐processing of tissue constructs. By closing feedback loops across all stages, AI can transform MIB into a safer, more precise, and more personalized therapeutic modality.

## 6 Discussion: Challenges and Pathways to Clinical Translation for Minimally Invasive Bioprinting

### 6.1 Fundamental Challenges of MIB

Although MIB holds transformative potential, its transition from benchside research to clinical reality is currently constrained by a multifaceted array of biological, technical, and socio‐ethical challenges.

A central barrier to clinical translation is the inherent biological complexity of internal organs [309], which requires not merely geometric filling but the restoration of multicellular organization and functional vascularization. To address the critical lack of vascular networks necessary for tissue survival, current strategies employ sacrificial printing techniques and bioinks designed to form vascular‐like structures; however, scaling these to match human physiology remains formidable. Consequently, a pragmatic translational pathway prioritizes stepwise clinical targets, advancing from the in situ deposition of acellular biomaterials or bioactive hydrogels to partial‐function constructs before attempting full organ‐mimetic regeneration. This process can be further de‐risked by integrating organ‐on‐chip and microfluidic platforms, enabling the controlled preclinical evaluation of maturation and safety before in vivo studies [289]. These biological goals are intrinsically linked to the printability of bioinks, where researchers are developing shear‐thinning formulations and triggered crosslinking mechanisms to ensure rapid stabilization post‐deposition [130]. Furthermore, composite bioinks incorporating nano‐structured scaffolds are being explored to reinforce mechanical properties and promote functional integration. Concurrently, the selection of cell sources presents a dilemma between the immune safety of autologous cells and the accessibility of allogeneic cells [115], necessitating rigorous informed consent protocols to manage patient expectations regarding these risks.

From an engineering perspective, achieving high‐fidelity printing is complicated by the dynamic nature of the in vivo environment, including dexterous pathways, soft tissue deformation, and physiological motions such as heartbeats and breathing. These factors, combined with the nonlinearities of robotic systems, specifically hysteresis, compromise precision. To mitigate these errors, feedback‐rich control systems are being developed to provide real‐time feedback by integrating orientation and tracking systems with pose and shape sensing technologies. Additionally, force and tactile feedback mechanisms are being incorporated to ensure safe interaction with surrounding tissues in confined spaces (see Section 4.6). These hardware solutions are increasingly augmented by AI‐driven control algorithms, which optimize printing parameters in real‐time to enhance printing accuracy and reproducibility. Crucially, these robotic platforms must operate under strict sterile conditions, requiring fabrication from medical‐grade materials compatible with standard sterilization protocols, such as autoclaving, gamma irradiation, or ethylene oxide, to prevent cross‐contamination and minimize the risk of surgical site infections [310].

Finally, the clinical adoption of MIB is obstructed by significant non‐technical barriers. The heavy reliance on patient‐specific imaging creates cybersecurity risks regarding the unauthorized use of digital anatomy, demanding strict data ownership laws [311]. Legally, the autonomous nature of robotic surgery introduces a liability dilemma, necessitating a new framework to define accountability boundaries between the surgeon and the device manufacturer in the event of errors. Economically, the technology remains capital‐intensive due to the cost of high‐end bioprinters, sterile culture environments, and the need for interdisciplinary teams. Without stringent commercialization oversight by regulatory bodies like the U.S. Food and Drug Administration (FDA), there is a risk that these costs could drive social inequality or foster illicit “black markets” for biofabricated organs, underscoring the need for policies that ensure equitable access and adherence to human rights standards.

### 6.2 From Preclinicals to Clinical Translation

Among global regulatory bodies, the guidelines established by the FDA serve as the most prominent and widely referenced benchmark for navigating this translational pathway. Central to this system are two approval pathways: the 510(k) Premarket Notification, which offers an expedited route for devices substantially equivalent to existing predicates, and the Premarket Approval (PMA), a rigorous process mandated for high‐risk or novel devices requiring comprehensive clinical evidence of safety and efficacy [312].

The regulatory trajectory for bioprinted constructs is stratified into three categories based on their biological and structural complexity. First, acellular scaffolds, such as those fabricated from decellularized extracellular matrix, are generally categorized as Class II medical devices (moderate risk) and may pursue the expedited 510(k) pathway, provided they demonstrate substantial equivalence to legally marketed predicate devices [313]. Second, as complexity increases to simple cellularized tissues containing bioinks and limited cell types (e.g., skin or cartilage), the regulatory burden intensifies. While specific simple analogues may qualify for 510(k) clearance if suitable predicates exist, the majority of cellularized constructs are typically classified as Class III medical devices [313], a category reserved for high‐risk devices that support or sustain human life, or present a potential unreasonable risk of illness or injury, necessitating the rigorous PMA process to ensure safety and effectiveness. Third, complex 3D‐bioprinted tissues and solid organs, which lack established predicates, must undergo the stringent PMA pathway to validate their physiological functionality and long‐term viability. This route entails a significantly longer timeline to complete the clinical trials required.

Regardless of the classification, the validation process necessitates a hierarchical progression from preclinical models to human trials. Initially, preclinical validation must be conducted in animal models, advancing from small animals (for proof‐of‐concept) to large animal models (e.g., porcine or ovine) that more accurately recapitulate human anatomy and physiology [314]. Only upon generating sufficient safety data can investigators apply for an Investigational Device Exemption (IDE) from the FDA to initiate human studies.

Clinical trials are subsequently executed in a phased approach. Early‐phase trials recruit small cohorts to evaluate initial safety and dosage parameters. Subsequent pivotal trials expand to larger patient populations to rigorously assess efficacy, compare outcomes against current standards of care, and monitor for adverse events. This process involves longitudinal surveillance to evaluate long‐term health impacts, such as immunogenic responses or graft stability [315]. Depending on the complexity of the construct, this regulatory journey, from initial design to final FDA market approval, is a multi‐year endeavor, often spanning decades for complex organs, to ensure that the technology is both safe and effective for widespread clinical adoption.

The reality of these extended timelines and the complexity of moving through such phases are clearly demonstrated by existing regenerative products. For instance, Epicel, an autologous cultured epidermal graft for severe burns, was approved by the FDA under the Humanitarian Device Exemption (HDE) in 2007, but its clinical evolution continued for years, with a subsequent label update via HDE Supplement 34 to include pediatric labeling, approved in February 2016[316]. More recently, the ReNew Hip Implant (CytexOrtho company), a bioabsorbable, highly porous implant designed to support cartilage repair, serves as a practical example of a technology currently in the early‐phase trials, has moved into early feasibility/first‐in‐human clinical evaluation to assess initial safety and performance [87, 317].

To conclude, the clinical success of MIB depends on addressing a multifaceted array of biological, technical, and socio‐ethical hurdles. Furthermore, these systems must successfully navigate stringent regulatory pathways to demonstrate absolute safety and efficacy. By integrating state‐of‐the‐art innovation with rigorous clinical protocols, MIB will fundamentally reshape the healthcare landscape, transforming personalized, on‐demand tissue repair into a clinical reality.

## 7 Conclusion

This review has systematically addressed the five core objectives outlined in the introduction, establishing a structured pathway for advancing Minimally Invasive Bioprinting (MIB). First, by delineating the evolutionary trajectory of in situ bioprinting, we highlighted the shift from open‐surgery approaches toward MIB as a key step for internal tissue repair. Second, we positioned soft robotics as a central hardware enabler, showing how inherent compliance and flexibility can support safer navigation and controlled deposition within confined anatomical environments that often challenge rigid systems. Third, through a comprehensive analysis of the six technology pillars, imaging, bioinks, soft actuation, sensing, modeling, and control, we identified the convergence required to achieve high‐fidelity in situ fabrication. Furthermore, our discussion of emerging directions suggests that 4D bioprinting, organ‐on‐a‐chip platforms, and artificial intelligence can enhance the adaptability, autonomy, and functionality of next‐generation bioprinting systems. Finally, our evaluation of technical and regulatory barriers underscores that, while clinical translation remains complex, it is achievable through rigorous validation and sustained interdisciplinary collaboration. In summary, MIB is a promising pathway toward patient‐specific regenerative interventions, and continued integration of these interdependent technologies is expected to accelerate progress from proof‐of‐concept demonstrations toward clinically reliable solutions.

## Conflicts of Interest

The authors declare no conflicts of interest.

## Data Availability

The authors have nothing to report.
